# Thermal Degradation Processes of Aromatic Poly(Ether Sulfone) Random Copolymers Bearing Pendant Carboxyl Groups

**DOI:** 10.3390/polym12081810

**Published:** 2020-08-12

**Authors:** Sandro Dattilo, Concetto Puglisi, Emanuele Francesco Mirabella, Angela Spina, Andrea Antonino Scamporrino, Daniela Clotilde Zampino, Ignazio Blanco, Gianluca Cicala, Giulia Ognibene, Chiara Di Mauro, Filippo Samperi

**Affiliations:** 1 Institute for Polymers, Composites and Biomaterials, IPCB-CNR, Via Gaifami 18, 95126 Catania, Italy; Concetto.puglisi@cnr.it (C.P.); emanuelefrancesco.mirabella@cnr.it (E.F.M.); angelaspina1@virgilio.it (A.S.); danielaclotilde.zampino@cnr.it (D.C.Z.); gcicala@unict.it (G.C.); filippo.samperi@cnr.it (F.S.); 2 Department of Civil Engineering and Architecture, University of Catania, Viale Andrea Doria 6, 95125 Catania, Italy; iblanco@unict.it (I.B.); giuliaognibene@live.com (G.O.); chiara.dimauro@hotmail.it (C.D.M.)

**Keywords:** poly(ether sulfones), diphenolic acid, thermal degradation mechanisms, DPMS, Py-GC/MS

## Abstract

Thermal degradation processes of poly(ether sulfone) random copolymers having different molar amount of diphenolic acid (DPA) units were studied by direct-pyrolysis/mass spectrometry, stepwise pyrolysis-gas chromatography/mass spectrometry and thermogravimetric techniques. Results highlighted that thermal degradation processes occur in the temperature range from 370 to 650 °C, yielding a char residue of 32–35 wt%, which decreases as the mol% of DPA units rises. The pyrolysis/mass spectra data allowed us to identify the thermal decomposition products and to deduce the possible thermal degradation mechanisms. Thermal degradation data suggest that the decarboxylation process of the pendant acid moiety mainly occurs in the initial step of the pyrolysis of the copolymers studied. Successively, the scission of the generated isobutyl groups occurs in the temperature range between 420 and 480 °C. Known processes involving the main chain random scission of the diphenyl sulfone and diphenyl ether groups were also observed.

## 1. Introduction

**Scheme 1:**
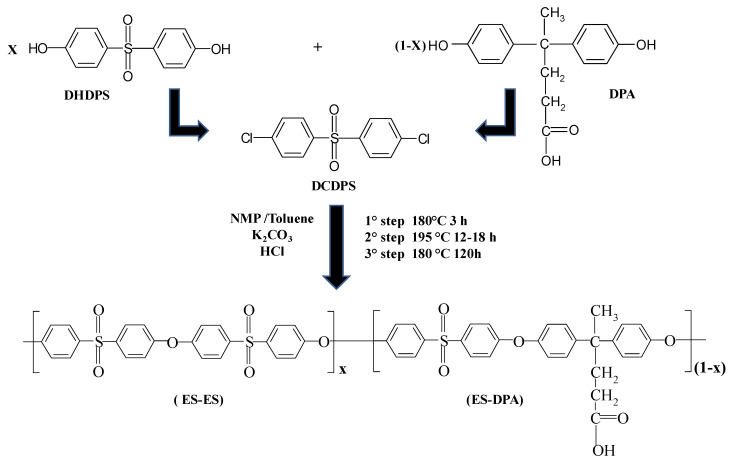
Schematic synthesis of P(ESES-co-ESDPA) copolymers.

**Scheme 2:**
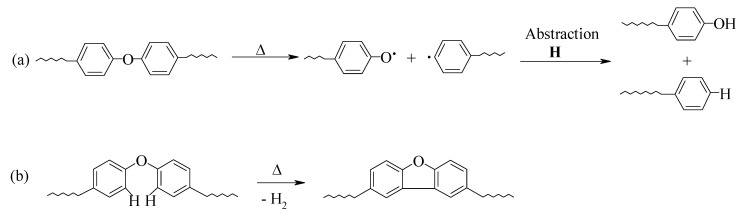
Thermal degradation mechanisms involving diphenyl ether moieties, (**a**) cleavage of the diphenyl ether bridges, (**b**) dehydrogenation process and formation of dibenzofurans.

**Scheme 3:**
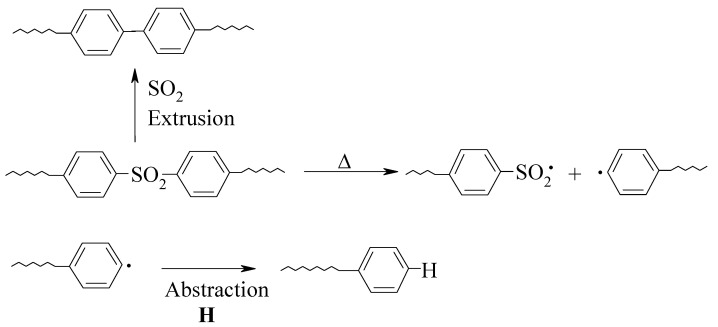
Thermal degradation mechanisms involving diphenyl sulfone units.

Aromatic poly(ether sulfones) (PESs) are thermoplastic materials of great engineering performance with high glass transition temperature, high chemical and thermal stability, good mechanical properties, and excellent resistance to hydrolysis and oxidation [1,2,3,4,5,6,7,8,9,10,11,12,13,14,15]. These polymers have aroused a remarkable scientific and industrial interest, founding applications as adhesives for metal-to-metal bonds [2], membranes for separating gases and solids from solution or isolation of proteins [3], matrices for fiber-reinforced composites, as toughening agents for thermosetting resins [5,6,7,8,9,10] and for the manufacture of optical waveguiding materials [11,12]. It has been reported [16,17,18,19,20,21] that a wide spectrum of thermal, chemical and mechanical properties, as well as morphological behavior, can be obtained by varying the type of monomer units in the corresponding copolymers or by derivatization of preformed commercial PESs. In this work, we report the studies of thermal degradation processes on a series of functionalized random copoly(ether sulfones) having pendant carboxylic acid groups. This type of functionalization is useful to solve a large applicability limit typical of PES, i.e., total hydrophobicity, improving the application capacity as membranes in the field of fuel cell, water purification, etc. It is expected that the insertion of hydrophilic pendant groups in the chain could modify the thermal properties of these materials [4,10,21]. In the present work, the thermal behavior of a series of copoly(arylene ether sulfones) containing different molar amounts of a structural unit with carboxylic pendant group was studied. All polymer samples were synthesized by solution polymerization via nucleophilic displacement polycondensation reaction between a stoichiometric amount of 4,4′-dichlorodiphenyl sulfone (DCDPS) and different molar ratios of 4,4-bis(4-hydroxyphenyl)pentanoic acid (known as diphenolic acid or biphenyl valeric acid, and indicated here as DPA), as schematized in Scheme 1 [22]. The synthesized random copolymers are referred therein as P(ESES-co-ESDPA). The thermal degradation behaviors of the copolymers were compared with those of the corresponding homopolymers: P(ESES) (commonly noted as PES) and P(ESDPA), synthesized in the same experimental conditions. As the knowledge of the degradation pattern of a polymer is an important step in choosing a material for product manufacture, the thermal degradation of either the homopolymers (PESs) [14,15,16,17,23,24,25,26,27,28,29,30,31,32,33] or copoly(arylene ether sulfone) (referred as PES-PPO) was extensively studied [13]. Considerable efforts have been made to reveal the thermal degradation mechanisms and the thermal degradation products by mass spectrometry [23,24,25,26,27,28,29,32,33], thermogravimetry [30] and gas chromatography [31]. In particular, the formation of pyrolysis products occurs at temperature higher than 400 °C due to the thermal cleavage of diphenyl ether and diphenyl sulfone moieties (Schemes 2 and 3) [13,23,24,25,27,28]. The elimination process of SO_2_ from the diphenyl sulfone bridges occurs at temperature above 450 °C, with the formation of products containing biphenyl units [13,23,24,25,27,28]. Pyrolysis products containing biphenyl and dibenzofuran units were also observed in the thermal degradation of PES-PPO copolymers at temperature higher than 500 °C [13,24]. Despite some previous studies on poly(ether sulfones) containing DPA units [16,17,18,19,34,35,36], until now, no detailed information on the thermal degradation mechanisms occurring during the thermal degradation of the P(ESDPA)-based polymers have been available in the literature. In this work, we aimed to obtain this information by direct-pyrolysis mass spectrometry (DPMS), stepwise pyrolysis-gas chromatography/mass spectrometry (Py-GC/MS) and thermogravimetric analysis (TGA). In particular, we used the DPMS method, as it allows the pyrolysis under high vacuum, at microgram level and adjacent to the electron emitter, so that almost-primary pyrolysis products can be detected and yield important information on the thermal degradation pathways of various classes of polymers [23]. The thermal degradation of (PES) and some random copoly(arylene ether sulfones) have been investigated by DPMS, and the pyrolysis data suggested the thermal degradation pathways sketched in Schemes 2 and 3 [13,24]. However, the DPMS spectra of complex polymeric structure often contain mixed information on the pyrolysis products, making the identification of the individual compounds rather difficult. The use of a complementary technique such as the stepwise Py-GC/MS method can allow the separation of individual thermal degradation compounds and permits the correlation of the structure of individual pyrolysate with the temperature of pyrolysis [26,28,32,33]. Combining DPMS, Py-GC/MS and TGA data, we identified the pyrolysis products, the thermal degradation mechanisms and the thermal stabilities of P(ESES-co-ESDPA) polymer samples. Their thermal behavior was compared with that of a known commercial PES (referred to as PESES) sample. This study on the thermal degradation of P(ESES-co-ESDPA) copolymers is of some fundamental importance to explore the field of application of these materials, including high temperature fuel cells, water purification, further structural modification, etc.

## 2. Materials and Methods

### 2.1. Materials

4,4′-dichlorodiphenylsulfone (DCDPS), 4,4′dihydroxydiphenylsulfone (DHDPS), 4,4-Bis(4-hydroxyphenyl) pentanoic acid, potassium carbonate (K_2_CO_3_), N-Methyl-2-pyrrolidone (NMP), toluene, tetrahydrofuran (THF) and methanol were purchased by Merch-Sigma Aldrich, Milan Italy. Before use, the reactive monomers (DCDPS, DHDPS and DPA) and K_2_CO_3_ were dried at 80 and 150 °C, under vacuum, for 24 h, respectively. All the other products were used without any treatment.

### 2.2. Synthesis

All tailored poly(ether sulfones) were synthesized from different amounts of DHDPS, DPA and stoichiometric amount of DCDPS by nucleophilic aromatic displacement, as schematized in Scheme 1. As an example, to synthesize the P(ESES-co-ESDPA) copolymer 50:50 mol/mol, DHDPS (10.89 g, 43.5 mmol) and DPA (12.45 g, 43.5 mmol) were solubilized in NMP (ratio 1:1,5 *w*/*v*) in a three-necked round flask equipped with a magnetic stirrer, a dropping funnel, a nitrogen inlet and a reflux condenser. Thereafter, 40 mL of toluene and a solution of K_2_CO_3_ (13.24 g, 95.8 mmol) in 50 mL of NMP were added, and the mixture was heated, under stirring, at 170 °C, until the water/toluene azeotrope distilled. An anhydrous NMP solution (50 mL) of the DCDPS monomer (25.0 g, 87.0 mmol) was added to the reaction mixture, and the temperature was raised and maintained at 180 °C for 3 h. After that, the reaction temperature was raised up to 195 °C and maintained at this temperature for 12 h; successively, the temperature was lowered to 180 °C, and the reaction was carried out for a further 12 h. After this step the reaction mixture was cooled down to the room temperature and a solution of HCl (12M) (10 mL) was added to protonate the pendant carboxyl groups. Finally, to obtain a fine light amber precipitate, the polymer was precipitated, drop by drop, into a 50:50 *v*/*v* water/methanol mixture (800 mL), filtered, washed several times with cold methanol and finally dried in an oven, at 110 °C, under vacuum (10 Torr), for 48 h. In order to remove the low-molar-mass (MM) oligomers, the crude polymers were further purified by continuous extraction in a Soxhlet apparatus. Tetrahydrofuran (THF), for either P(ESES) homopolymer and P(ESES-co-ESDPA) copolymers with a molar composition of ESDPA units from 10 to 30 mol%, and ethyl ether, for the copolymers with an ESPDA molar composition higher than 50%, since these ones are soluble in THF, were used. Crude and purified copolymers were thermally analyzed by TGA and Py-MS techniques, and they were characterized by ^1^H-NMR and FTIR methods. In Table 1, some data of the purified copolymers and homopolymers studied are summarized. The ^1^H-NMR and FTIR spectra of the P(ESES-co-ESDPA) 50/50 copolymer are displayed in Supplementary Materials Figure S1a,b, respectively. Both spectra confirm the formation of the copolymer sample. The complete characterization of all copolymers is discussed in detail in another specific work [37]. Some relevant data, such as molar composition, molar mass and yield of polymerization, are summarized in Supplementary Materials Table S4, which displays that the molar compositions agree with the feed data. Furthermore, the average weight molar mass (Mw) of each sample, ranging from 14 to 20 kg/mol, and the number average molar mass (Mn), ranging from 6.15 to 8.0 kg/mol, are reported. Average molar masses were measured by SEC analysis, using DMF as eluent and applying the calibration curve build, using polystyrene narrow standards. Mn values were also calculated through 1H-NMR analysis, and the values agree with the SEC ones (Supplementary Table S4, [37]).

### 2.3. Methods of Analysis

**Figure 1:**
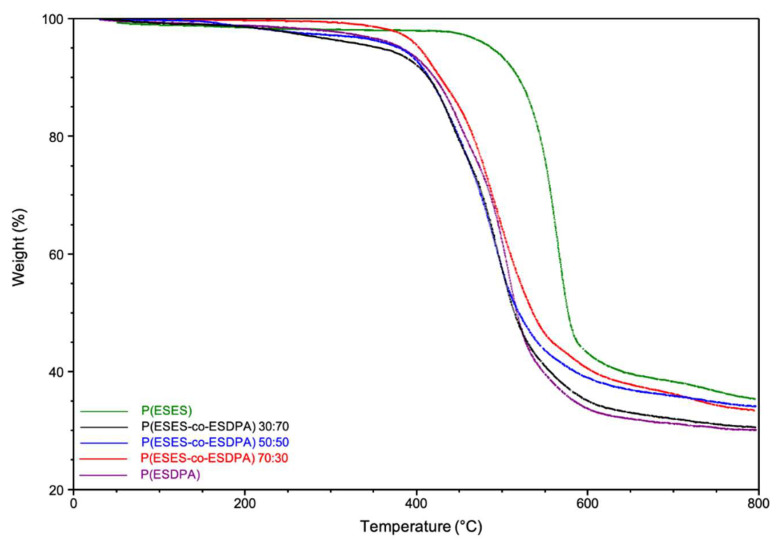
Overlay of thermogravimetric curves of P(ESDPA)-co-(ESES) copolymers and their homopolymers.

**Figure 2:**
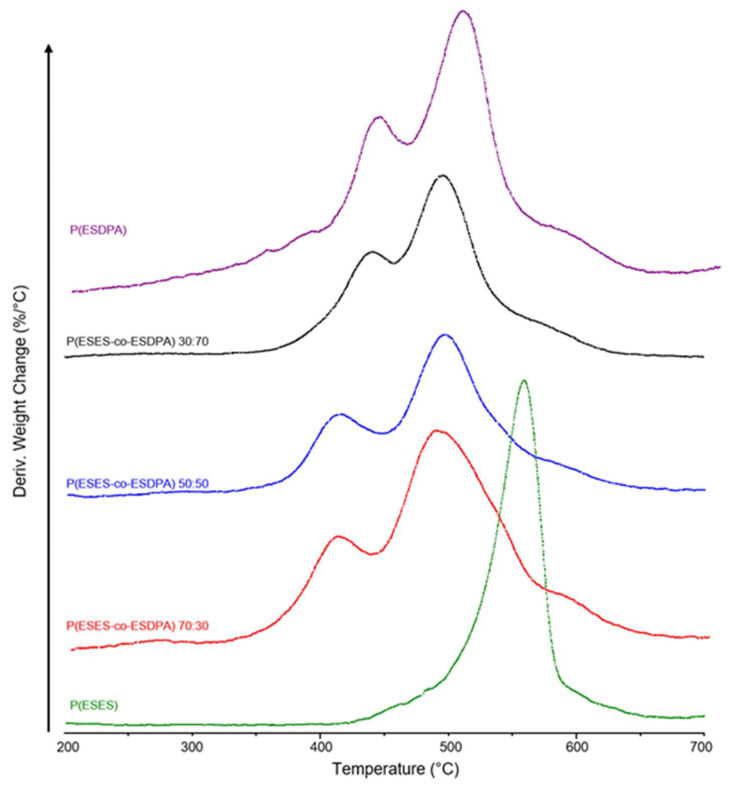
Differential thermogravimetric (DTG) curves of the P(ESDPA)-co-(ESES) copolymers and the corresponding homopolymers. DTG curves are shifted vertically for clarity.

The thermal stability of the whole set of polymers was investigated by TGA (TA Instruments Q500, New Castle, Delaware, US), under nitrogen flow (60 mL/min), at a heating rate of 10 °C/min, from 50 to 800 °C, using 2 ± 0.1 mg of sample. Instrument temperature and weight were calibrated by using high-purity magnetic standard for the Curie temperature and some exact weights, respectively, following the calibration procedure by the instrument control software. The TGA data were analyzed by the TA instruments operating software (New Castle, Delaware, US). For each sample, both weight loss and weight-loss rate vs. temperature were determined (Figure 1 and Figure 2). Furthermore, the initial decomposition temperatures (*T*_i_), the half decomposition temperatures (*T*_1/2_) and temperature at the maximum rate of the weight loss (*T*_max_) for the single stage observed in the TGA traces were evaluated.

Direct-introduction mass spectrometry analysis (DPMS) was carried out on a triple quadrupole mass spectrometry detector TQ8040 (Shimadzu Corporation, Kyoto, Japan) with an electronic ionization of 70 eV. All polymer samples were pyrolyzed in the standard direct insertion probe for solid material, heated from 30 to 500 °C at a heating rate of 10 °C/min and maintained at this temperature for 20 min. Electron impact mass spectra (70 eV) were recorded continuously at a scan rate of 500 amu/s (atomic mass unit). Mass spectra were elaborated by means of the GC/MS solution software (Shimadzu Corporation, Kyoto, Japan).

The Py-GC/MS experiments were carried out with a Multi-Shot Pyrolyzer (EGA/PY-3030D, Frontier Labs, Saikon, Koriyama, Fukushima, Japan), coupled to a GC system GC-2020 (Shimadzu Corporation) linked to a triple quadrupole mass spectrometry TQ8040 (Shimadzu Corporation) (electronic ionization 70 eV). The gas chromatography apparatus was equipped with Ultra Alloy^®^ Metal Capillary Column (Frontier Labs, Japan, stationary-phase 5% di-phenylmethylpolysiloxane, with an inner diameter of 250 µm, a film thickness of 0.25 µm and a length of 30 m). Interfaces of Py-GC and GC/MS were kept at 300 and 250 °C, respectively. About 0.1 mg of each polymer sample was placed, without pretreatment, in the crucible (quartz capillary sample holder) and was pyrolyzed at fixed temperatures: firstly at 400 °C, then at 500 °C and finally at 600 °C, in order to characterize the pyrolysis product formed at different temperatures. The GC separation was obtained by applying the following temperatures program: isotherm at 50 °C for 1 min, heating from 50 to 100 °C at 30 °C/min, isotherm at 100 °C for 5 min and heating from 100 to 300 °C at 10 °C/min and finally isotherm at 300 °C for 10 min. The carrier gas was helium at a controlled flow of 1.78 mL/min. The split ratio was 1/10 of the total amount of carrier gas. Mass spectra were recorded under electron impact ionization energy at 70 eV, and the flow rate was kept constant. The MS detector was scanned from 30 to 500 *m*/*z*, at a scan rate of 2500 scans. Before each analysis, blanks were carried out by placing the crucible empty in the furnace and applying the same pyrolysis program (see above). Data analyses were performed with a LabSolution GC/MS Analysis software (Shimadzu Corporation, Kyoto, Japan).

Several pyrolysis products were identified by using library mass spectra (NIST 14 and Wiley database), accepting matches ≥ 85%. In case of ambiguity, the evolved products were identified by taking into account the DPMS data and also studying the fragment ions observed in the recorded mass spectra. ^1^H-NMR and ^13^C-NMR spectra of the whole set of synthesized homo and copolymers were obtained by a Bruker Advance 400 spectrometer. The analyses were performed at 50 °C at 400.13 MHz for protons and at 100 MHz for carbon analyses. Samples were dissolved in deuterated dimethyl sulfoxide (DMSO-d_6_), at the concentration of 25 mg/mL.

## 3. Results and Discussion

### 3.1. Thermogravimetry

Data on thermal stability of all poly(ether sulfones) studied by TGA are summarized in Table 1. It can be seen that the stability of all carboxylic acid functionalized P(ESES-co-ESDPA) copolymers, as well as of the P(ESDPA) homopolymer, is lower than that of the hydrophobic P(ESES) homopolymer.

The characteristic TGA and differential thermogravimetric (DTG) curves of some P(ESES-co-ESDPA) random copolymers and of their homopolymers are displayed in Figure 1 and Figure 2. All copolymer samples degrade in the 370–680 °C temperature range, with the maximum rate of weight loss (*T*_max_) that depends on the copolymer composition. The *T*_max_ value decreases as the molar amount of the DPA units in the copolymer increases, reaching that of 495 °C for the P(ESDPA) homopolymer. The TGA curves show the other two degradation peaks, at about 380–450 °C and 570–600 °C, that appear as shoulders on the DTG curves (Figure 2). In particular, in agreement with the literature data, the TGA curve of the P(ESES) homopolymer shows the *T*_max_ at 566 °C and another little delineated maximum at 590 °C that appears as a shoulder in DTG curve (Figure 2), yielding a char residue of 35.4%.

The TGA curve of the P(ESDPA) sample shows two well-defined degradation steps with the maxima at 437 and 495 °C, whereas the maximum observed as a shoulder at about 590 °C is low definite. This homopolymer yields a char residue of about 32.4%. Comparing the TGA curves of both homopolymers, we hypothesize that the first thermal-degradation step of the P(ESDPA) polymer, occurring in the temperature range from 380 to 470 °C, should be due to the degradation mechanisms involving the pentanoic acid (valeric acid) moiety belonging to the DPA units. From the TGA and the DTG curves of the P(ESES-co-ESDPA) copolymers that emerge, we can see that their thermal stabilities lie between those of the two corresponding homopolymers. The copolymers yield char residues ranging from 33.4 to 34.5%, at 800 °C (Table 1). Char residues of replicate samples gave values very similar (standard deviation ± 0.1), indicating the purity of the homopolymers and copolymers synthetized. The char residue decreases as the mol% of the ESDPA molar units in the copolymer chains increases, suggesting that the decomposition processes occurring at temperatures lower than 450 °C could depend on these sequences. The analysis of the onset temperatures (*T*_i_) highlights a significant decrease (95–105 °C) of the P(ESES) homopolymer *T*_i_ as the molar amount of the DPA units in the copolymers increases. This decrease was particularly evident for the crude P(ESES-co-ESDPA) 70:30 and P(ESES-co-ESDPA) 30:70 copolymers, showing temperature differences of about 110 and 102 °C, most probably due to the presence of low-molar-mass oligomers, as confirmed by their further purification by selective solvent extraction. The thermogravimetric data summarized in Table 1 concern the accurately purified polymers. The half-decomposition temperatures (50% weight loss) do not reach the relevant decrease observed for *T*_i_ values, display a decrease range of 27–62 °C and show a degradation behavior similar to that of the major degradation process (*T*_max_).

### 3.2. Direct Pyrolysis in Mass Spectrometry

**Figure 3:**
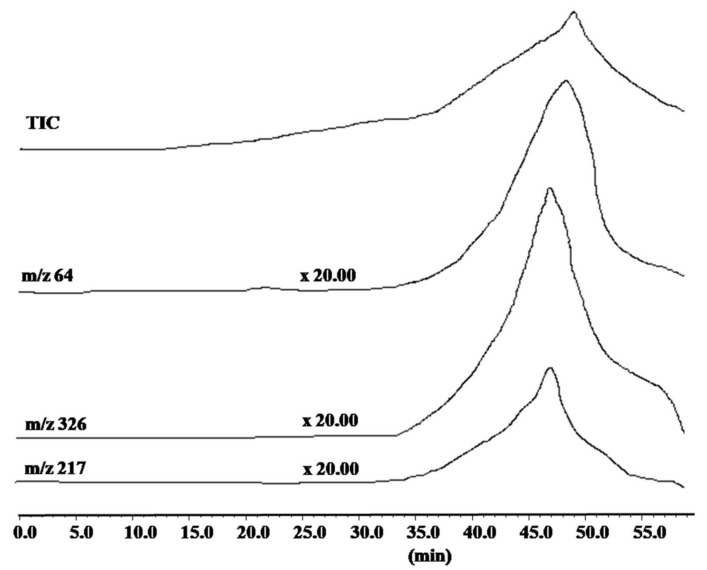
Total ion current (TIC) trace and single ion curves (SIC) of the ions at *m*/*z* 64, 326 and 217 evolved in the EI-DPMS of the P(ESES) homopolymer.

**Figure 4:**
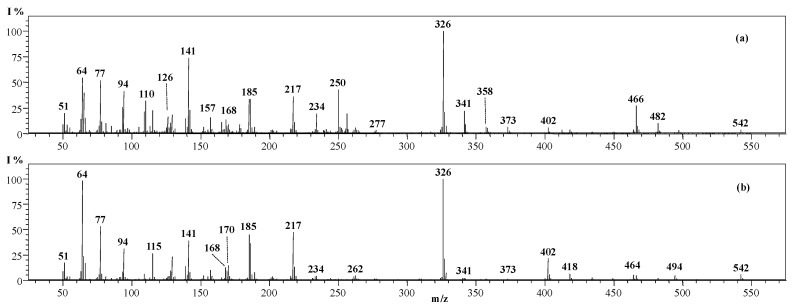
EI-DPMS spectra of the P(ESES) sample recorded in the temperature ranges (**a**) 430–480 °C and (**b**) 480–500 °C.

In order to obtain more detailed information on the thermal degradation products and to shed more light on the thermal degradation mechanisms that generate them, all samples were studied by the DPMS method described in the experimental section. The results were compared with those obtained in some similar studies carried out for the PES homopolymer [24] and the copoly(arylene ether sulfones) [13]. Two factors are assessed in the studies concerning the thermal degradation mechanisms of polymers: (1) the specific bond dissociation energies of the chemical groups composing the main chains, the side groups and the end groups; and (2) the stability of the formed radicals. Furthermore, in many cases, the reactivity of the end groups (e.g., OH, NH_2_, COOH, etc.) is also considered [23]. For P(ESES-co-ESDPA) polymers, the chemical bond dissociation energies (BDE) of the different linkage in the polymer backbone are as follows [26,38,39,40]: –C–COOH bond and consequent CO_2_ scission, BDE = 66–71 kcal/mol; C–S bond, BDE = 55–60 kcal/mol; C–C bond, BDE = 80–85 kcal/mol; C–H bond, BDE = 90–100 kcal/mol; C–O bond, BDE = 86.1 kcal/mol. Therefore, on the basis of BDE values, the order of polysulfones bond breaking (weakest to strongest) can be the following: –C–COOH, Ph–SO_2_, Ph–O, Ph–C(CH_3_)–CH_2_, C–(CH_3_), C–CH_2_ and Ph–H. We believe that DPMS analysis gives pertinent information about the role of each bond in the pyrolysis pathways of the functionalized polysulfones studied. Figure 3 displays the total ion current (TIC) of the purified P(ESES) homopolymer, and the temperature SIC (single ion current) of diagnostic ions (i.e., *m*/*z* 64, 326 and 217) recorded by the EI-DPMS method. The EI (70 eV) mass spectra recorded at a probe temperature ranging from 430 to 480 °C and from 480 to 500 °C are displayed in Figure 4a,b, respectively. The relevant structural assignments of the observed molecular ions and fragments ions are summarized in Table 2.

The mass spectra in Figure 4 show peaks at *m*/*z* 542, 482, 466, 402, 326, 250 and 234, corresponding to the molecular ions containing ether-sulfone (ESES) sequences, and terminated with H and/or OH groups. The species at *m*/*z* 482, 466, 250 and 234 can be generated by the main chain scission of the diphenyl ether units (Scheme 2), whereas the molecular ions at *m*/*z* 542, 326 and 402 are formed by the concomitant scissions of the Ph–O (Scheme 2) and Ph–SO_2_ (Scheme 3) bonds along the macromolecular chains. The mass peak at *m*/*z* 217 corresponds to a fragment ion generated by the homolytic cleavage of the diphenyl ether bridges (Scheme 2). The pyrolysis product at *m*/*z* 464 evolved at a temperature higher than 480 °C (Figure 4b) and contains a dibenzofuran moiety in the backbone (Table 2); it can be formed in accordance with Scheme 2b. The mass peaks at *m*/*z* 494, 418, 402 and 262 correspond to the pyrolysis products having biphenyl moiety which are formed by SO_2_ (*m*/*z* 64) elimination (Scheme 3). The pyrolysis products having biphenyl moiety are formed only at later times than that of the corresponding species that contain the initial SO_2_ units. The evolution of SO_2_ (*m*/*z* 64) occurs in the temperature range 400–500 °C, the SIC curve is isochronous with the TIC curve. The presence of thermal degradation products at *m*/*z* 186, 418 and 494 indicate that structural rearrangements occurred during the pyrolysis process, as already observed [24].

**Figure 5:**
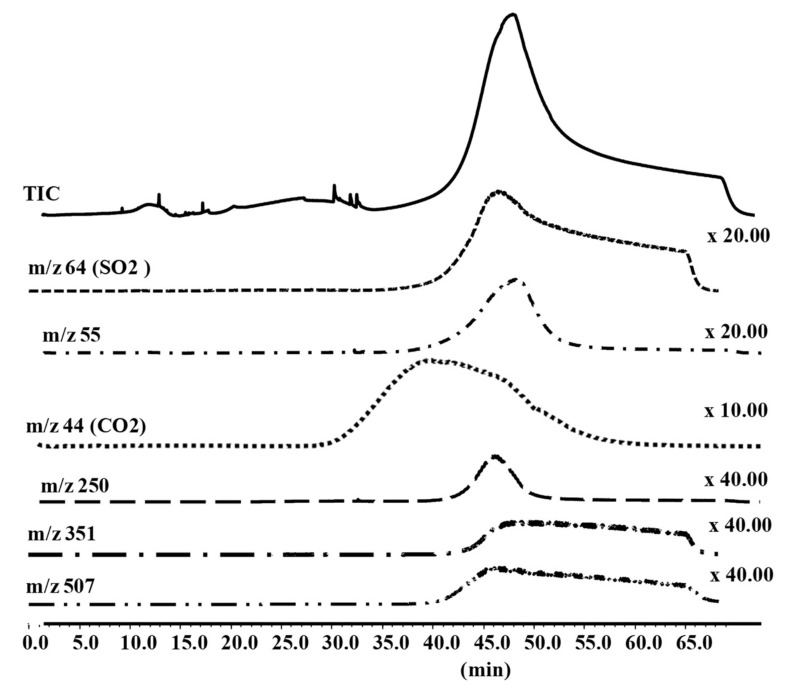
TIC trace and single ion curves (SIC) of the ions at *m*/*z* 44, 55, 64, 250, 351 and 507 evolved in the EI-DPMS of the P(ESDPA) homopolymer.

**Scheme 4:**
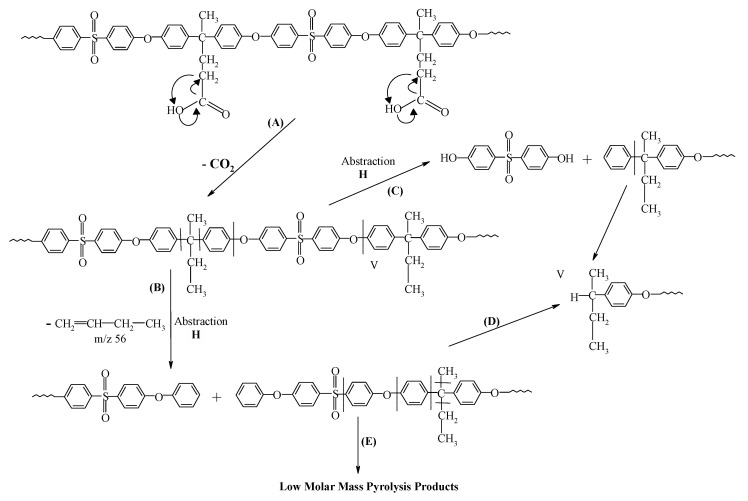
Thermal-degradation mechanisms involving the ESDPA sequences: (**A**) decarboxylation process; (**B**) multiple cleavage of phenyl-alkyl (Ph–C) bonds; (**C**) scission of phenyl-ether (Ph–O) bonds; (**D**) cleavage of Ph–O and Ph–C bonds; (**E**) cleavage of alkyl-alkyl (C–C) bonds, phenyl–O bond and phenyl–SO_2_ bond.

Our results are in agreement with those of the literature that were obtained by different DPMS apparatus and experimental conditions [13,24]. Figure 5 shows the TIC and SIC curves of the ions at *m*/*z* 44, 55, 64, 250, 351 and 507 recorded during the DPMS of the P(ESDPA) homopolymer. The SIC curve of the ions at *m*/*z* 44 indicates that the decarboxylation process (Scheme 4) occurs in the initial stage of the pyrolysis of the P(ESDPA) homopolymer, in accord with our hypothesis based on the BDE values. CO_2_ molecules are generated and evolved in the temperature range 350–470 °C. The CO_2_ loss was observed through TG–MS study of polysulfone homopolymer having COOH side group (26).

**Figure 6:**
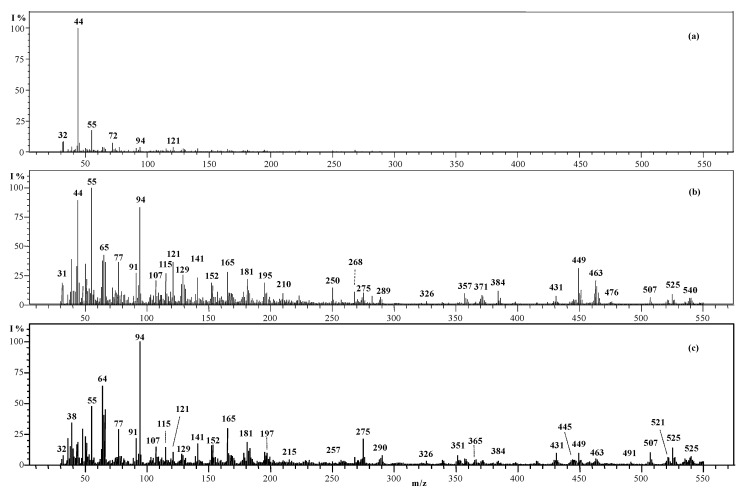
EI-DPMS mass spectra of the P(ESDPA) polymer recorded in the temperature ranges (**a**) 380–430 °C, (**b**) 430–480 °C and (**c**) 480–500 °C.

The EI (70 eV) mass spectra recorded in the temperature ranges 380–430 °C, 430–480 °C and 480–500 °C are shown in Figure 6, and the relevant assignments are reported in Table 2. The EI (70 eV) mass spectrum, recorded in the temperature range 380–430 °C (Figure 6a), shows an intense peak at *m*/*z* 44 (CO_2_), confirming that the early stage of the thermal decomposition of P(ESDPA) sample is due to the decarboxylation process of the DPA units (Scheme 4, route A). Successively to the CO_2_ loss process, the specific fragment at *m*/*z* 55 was generated by the scission of both Ph–C bonds belonging to the modified DPA units (Scheme 4, route B). The concomitant multiple cleavage of either phenyl-alkyl (Ph–C) and phenyl-ether (Ph–O) bonds allows the formation of the fragment ions at *m*/*z* 326 and 129, respectively (Scheme 4, route B). Moreover, the random multiple cleavage of phenyl-ether (Ph–O) and alkyl-alkyl (C–C) bonds induces the formation of the fragment ions at *m*/*z* 521, 491, 431, 289, 275, 210, 197, 195 and 181. The formation of specific fragments at *m*/*z* 540, 525, 507, 365 and 351 having biphenylene units along the chains also involves the SO_2_ (*m*/*z* 64) elimination process (Scheme 3). In particular, the fragment ions at *m*/*z* 365 and 351 develop at temperatures higher than the other ones.

**Scheme 5:**
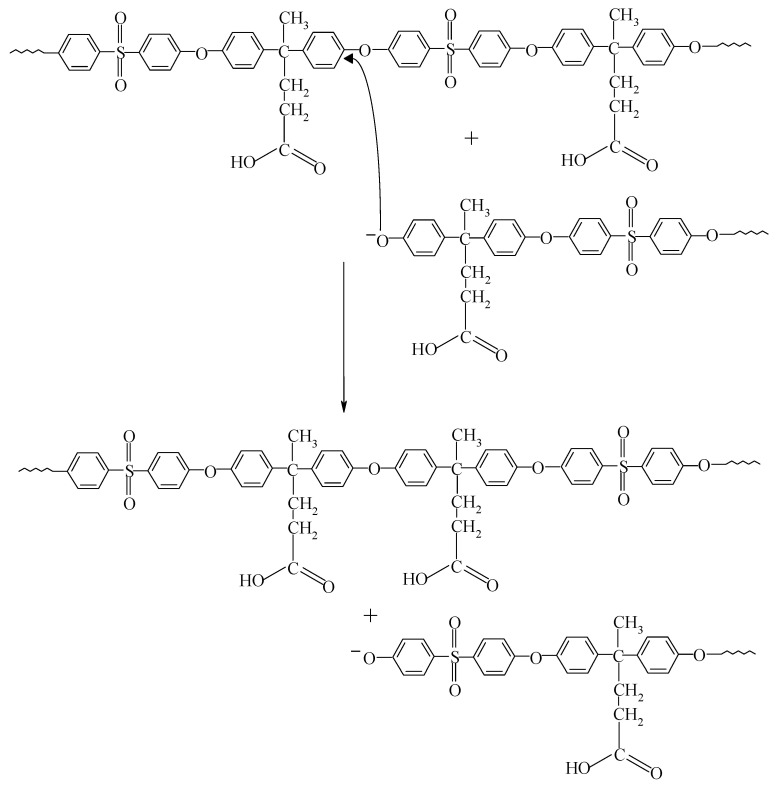
Trans-etherification reaction that generates the DPA–DPA homo-sequences along the P(ESES-co-ESDPA) copolymer chains.

The presence of the ions products at *m*/*z* 449 and 463, corresponding to pyrolysis products having dibenzofuran moieties (Table 2), suggests that the dehydrogenation process, which allows their formation (Scheme 2b), occurs at a temperature lower than that observed for P(ESES) one, probably due to the major flexibility of the phenyl-alkyl moiety of the modified DPA units. The fragments ions at *m*/*z* 384, 371 and 357 are assigned to the fragment ions generated from to unexpected alternated DPA–DPA sequences (phenyl-alky-phenyl-ether-phenyl-alkyl-phenyl-ether). These homogeneous sequences can be generated by trans-etherification reactions (Scheme 5) occurring during the synthesis or the pyrolysis process. Contrary to the P(ESES) homopolymer pyrolysis behavior, in the case of P(ESDPA), the pyrolysis products containing the intact ESDPA repeat unit (mass 500.2 Da) were not observed, owing to the quantitative elimination of CO_2_ and the further scission processes involving the diphenolic acid units (Scheme 4).

**Figure 7:**
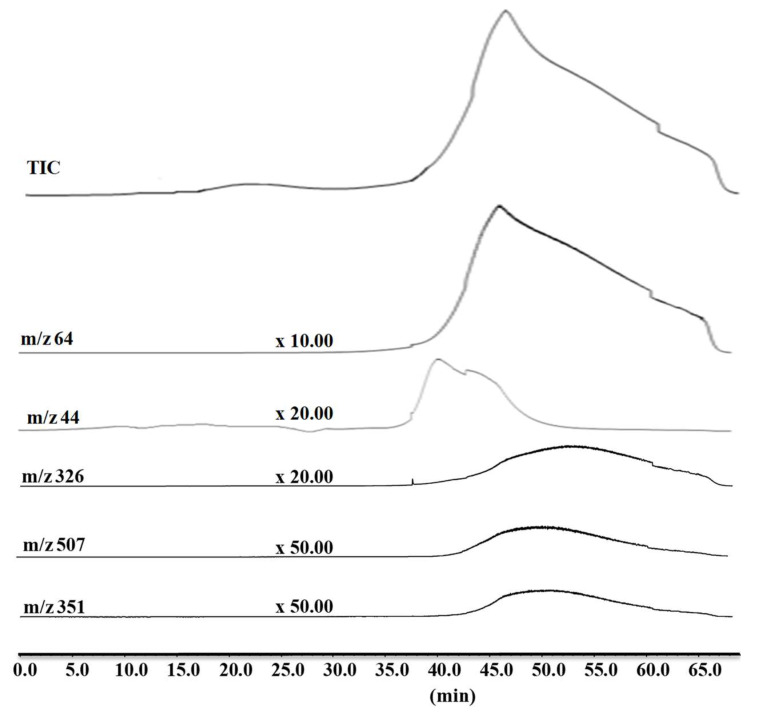
TIC trace and SIC curves of the ions at *m*/*z* 44, 64, 326, 351 and 507 recorded during the EI-DPMS of the random copolymer P(ESES)-co-(ESDPA) 50/50.

**Figure 8:**
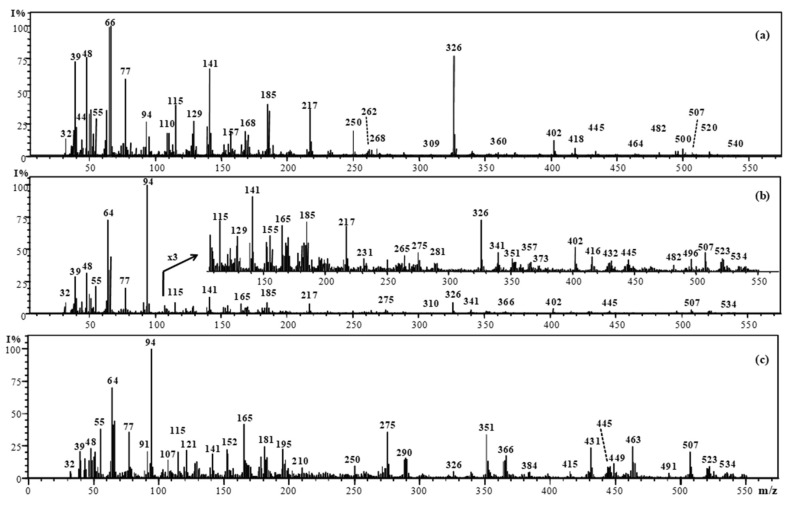
EI-DPMS spectra of the P(ESES)-co-(ESDPA) random copolymers (**a**) 70/30, (**b**) 50/50 and (**c**) 30/70 recorded in the temperature range 480–500 °C.

However, some peaks as those at *m*/*z* 540, 525, 507 and 431 are diagnostic for the ESDPA sequence. Taking into account the pyrolysis pathways occurring for the homopolymers (samples 1 and 7), we think that all processes compete during the thermal degradation of the P(ESES-co-ESDPA) copolymers. The influence of each mechanism should depend on the ESES/ESDPA molar ratio. In particular, we hypothesize that the decarboxylation process is responsible for the initial degradation of all copolymers, as confirmed by the TIC and SIC curves of the ions at *m*/*z* 44, 64, 326, 351 and 507 recorded during the DPMS analysis of the random copolymer P(ESES-co-ESDPA) 50/50 (Figure 7). The same behavior was observed for all copolymers studied. The relative intensity of the mass peaks, corresponding to the different fragment ions, depends on the molar composition of the ESES and ESDPA sequences in the copolymer, as shown in the EI (70 EV) mass spectra of the copolymers 70/30, 50/50 and 30/70, recorded in the temperature range 480–500 °C (Figure 8).

**Figure 9:**
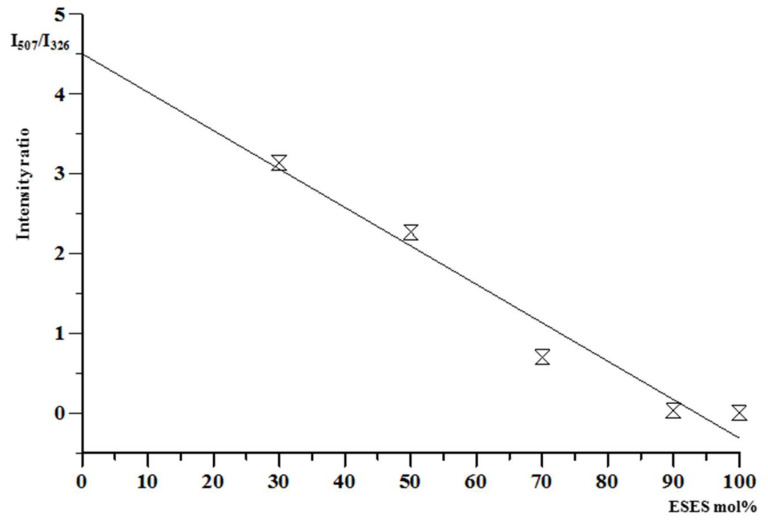
Evolution of the relative intensity of peak ions at *m*/*z* 507 and 326 as a function of the ES–ES mol% in the copolymers.

The mass spectra show typical fragment ions at *m*/*z* 44, 55, 275, 431, 445 449, 507, 521, 525 and 540, produced by the thermal degradation of the ESDPA units, and others at *m*/*z* 482, 402, 326, 217 and 185, generated by the thermal fragmentation process involving the ESES sequences. Some peaks (Figure 8), referable to the different amounts of ESES and ESDPA units along the chains, show different relative intensities as the molar composition of the copolymer changes. In particular, the intensity of the ion peak at *m*/*z* 507 (I_507_), due to the ESDPA moiety, decreases as the molar composition of the ESES units in the copolymers increases, whereas that of the ion at *m*/*z* 326 (I_326_), typical for the ESES sequences, increases. Figure 9 shows the intensity ratio change of the ions at *m*/*z* 507 and 326 as a function of the ESES mol%.

The intensities of these ions were calculated from the EI-DPMS mass spectra of the copolymer samples by averaging at least 30 independent scans relative to the mass spectra of the products evolved in the temperature range 450–500 °C, obtaining an excellent fitting (standard deviation of about ± 3%). Their relative intensity is in agreement with the following linear equation: Y = 4.5061 − 0.0482073X. Therefore, reliable information on the molar composition of unknown P(ESES-co-ESDPA) copolymers could be deduced by measuring the relative intensity of the ions at *m*/*z* 507 and 326, in the mass spectra recorded in the temperature range 450–500 °C.

The mass spectra recorded by the DPMS method show the mixtures of the primary degradation products and of their fragment ions, as a function of the pyrolysis temperature. However, this method does not permit the separation of each pyrolysis product. Therefore, in order to differentiate the pyrolyzed gaseous products in specific temperature regions, and to record the mass spectra of each product formed, we applied the complementary stepwise Py-GC/MS technique.

### 3.3. Py-GC/MS

For these experiments, the temperatures chosen were 500 and 600 °C, which are slightly higher than the temperatures of the peaks of the DTGA and 400 °C, to verify the formation of CO_2_ highlighted by the DPMS experiment.

The relevant structural assignments of the most important gaseous pyrolysis products observed by Py-GC/MS analysis of the polysulfones are summarized in Table 3 and Table 4.

**Figure 10:**
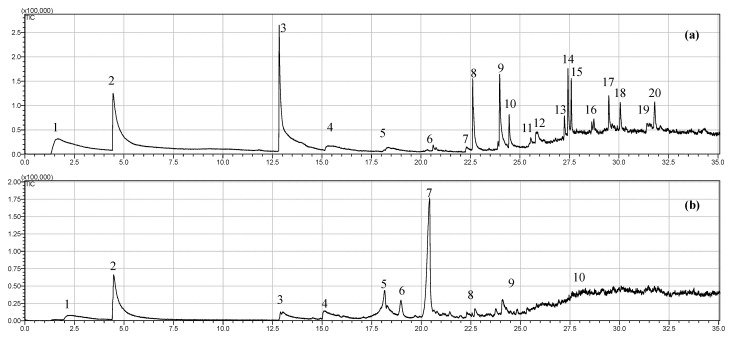
Py-GC/MS pyrograms P(ESES) detected at (**a**) 500 and (**b**) 600 °C.

Py-GC/MS data obtained for the P(ESES) homopolymer are in accordance with the literature [27,28] and the DPMS (see above) ones [13,23,24,25,26]. Figure 10 shows the pyrograms recorded at 500 and 600 °C. We identified the pyrolysis products by mass spectra and confirmed them by their comparison with those of model compounds, also using the literature data and the DPMS data reported above. The major pyrolysis products identified are in agreement with the degradation mechanisms displayed in the Scheme 2 and Scheme 3. The structures, retention times and major mass fragments of the pyrolysis evaluated by Py-GC/MS of P(ESES) at 500 and 600 °C are shown in Supplementary Materials Table S1. Supplementary Figure S2 displays the pyrogram and the EI mass spectra of the P(ESES) pyrolyzed at 400 °C. Supplementary Materials Figures S3 and S4 report the EI (70 eV) mass spectra corresponding to each peak in the chromatograms shown in Figure 10. At 500 °C (Figure 10a), several pyrolyzed gaseous products are observed, and peaks 1–5 and 9 are reported in the Table 3. Pyrolysis products bearing biphenyl moiety formed by elimination of SO_2_ (Scheme 3), peaks 7, 8, 10, 11, 33, 17 and 20, are ascertained (Table 3). Pyrolysis products, due to the fragmentation processes involving the diphenyl ether groups (Scheme 2), i.e., peaks 8, 10, 11, 13, 14,15,16 and17, are also detected (Table 3). The pyrolysis product at *m*/*z* 344 (peak 16) indicates that some polymer chains are terminated with 4-chloro diphenylsulfone moiety. In comparison with the published Py-GC/MS data [27,28], in our experimental condition, the pyrolysis products with a mass higher than 320 Da were evaluated. The plot of the pyrolysis products generated at 600 °C is shown in Figure 10b. The corresponding mass spectra reveal the formation of benzene (peak 1), phenol (peak 2), 4-hydroxyl diphenyl (*m*/*z* 170, peak 3) and dibenzofuran (peak 4). Moreover, the pyrolysis products generated by multiple bond-breaking reactions (Scheme 2 and Scheme 3) were identified such as that at *m*/*z* 402 (peak 5). The other peaks observed are due to the fragmentation and rearrangement processes involving the pyrolysis products with very high molar mass. Py-GC/MS data obtained at 400 °C (Supplementary Materials Figure S2) revealed the formation of few pyrolysis products, which may be due to the fragmentation of the end chains.

**Figure 11:**
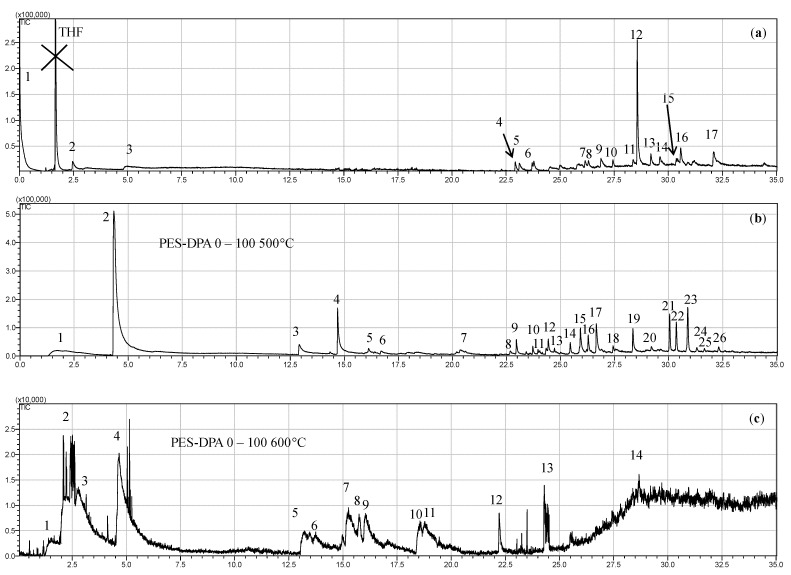
Py-GC/MS pyrograms P(ESDPA) taken at (**a**) 400, (**b**) 500 and (**c**) 600 °C.

The Py-GC/MS pyrograms from P(ESDPA) homopolymer obtained at 400, 500 and 600 °C are shown in Figure 11. The Py-GC/MS data are summarized in Supplementary Materials Table S2, and the corresponding mass spectra are shown in Supplementary Materials Figures S5–S7, in the order of increasing retention time for each pyrolysis temperature. The first peak of the chromatogram at 400 °C (Figure 11a), at very low retention time, confirms the formation of CO_2_ in the initial degradation step, in accordance with the Scheme 4 (route A) and the DPMS data discussed above to. Toluene (peak 2) and phenol (peaks 3) are formed at this temperature. The decarboxylation process leads to the formation of the 4,4′-diphenoxy 2-isobutyl units along the chains. Therefore, the pyrolysis products of the P(ESDPA) sample should be generated by the several degradation reactions which involve the different bonds along the polymer chains, as sketched in Scheme 2, Scheme 3 and Scheme 4. The concomitant breaking of the phenyl-sulfone bonds (Scheme 3) and the diphenoxy isobutyl units (Scheme 4) allows the formation of the products at *m*/*z* 290 (peak 8), 324 (peak 11) and 344 (peak 12). The pyrolysis products at mass 310 Da (peak 10) and 324 Da (peak 11) are diagnostic of the diphenyl sulfone units linked to the DPA units. Other peaks are due to either thermal and EI fragmentation of the pyrolysis products at higher molar mass. In comparison, the chromatogram recorded at 500 °C (Figure 11b) shows more peaks than that at 400 °C. At 500 °C, besides the elimination of either SO_2_ (peak 1, Figure 11b) and phenol (peak 2), the formation of the pyrolysis products generated by the concomitant degradation mechanisms sketched in Scheme 2, Scheme 3 and Scheme 4 is observed. The pyrolysis products bearing the dibenzofuran moiety, such as those at mass 288 (peak 11b) and 364 (peak 23), confirm that these units are formed (Scheme 2b) at temperature higher than 400 °C. The pyrolysis products at masses 198, 260, 272, 274, 288, 286, 306, 304, 355, 324, 352 and 366 (see Supplementary Materials Table S2) are formed owing to the different breaking bonds of the pre-generated diphenyl-isobutyl units along the chains (Scheme 5), which occur in parallel to the typical scissions of the diphenyl ether (Scheme 2) and diphenyl sulfone units (Scheme 3). Furthermore, the product at mass 355 Da (peak 18, Supplementary Materials Table S2) confirms that DPA–DPA homo-sequences can be generated by transetherification reactions (Scheme 5) during the heating of the P(ESDPA) polymer, as observed by DPMS studies (see above). At 600 °C (Figure 11c), the pyrolysis of P(ESDPA), as well as of the P(ESES) homopolymer, leads to the formation of SO_2_, benzene, toluene, 4-hydroxy diphenyl and dibenzofuran, which are typical pyrolysis products generated by the thermal degradation mechanisms involving diphenyl-ether/diphenyl-sulfone (ES) units. Moreover, the evolution of fluorene products (peaks 9 and 12, Figure 11c) was observed owing to the recombination reactions involving the generated aliphatic–aromatic sequences, which can occur at high temperatures. The product at mass 184 (peak 10, Figure 11c, and Supplementary Materials Table S2) is formed by degradation of the generated diphenyl isobutyl–phenyl ether units (Scheme 5). Furthermore, the unknown pyrolysis fragments observed at retention times higher than 22.5 min (Figure 11c and Supplementary Materials Table S2) could form owing to the electron impact fragmentation of the pyrolysis products having molar mass higher than 500 Da.

**Figure 12:**
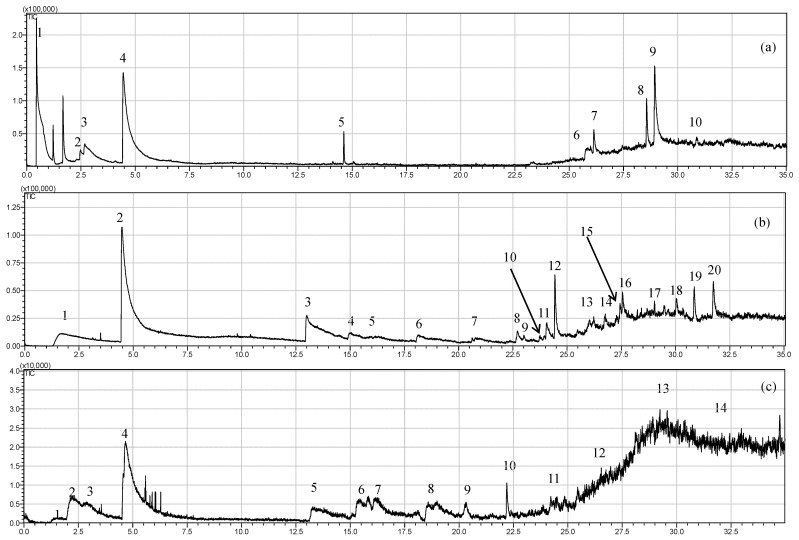
Py-GC/MS pyrograms P(ESES)-co-(ESDPA) 50:50 copolymer recorded at (**a**) 400, (**b**) 500 and (**c**) 600 °C.

The Py-GC/MS results recorded for the P(ESES-co-ESDPA) copolymers were analyzed, taking into account either the thermal degradation mechanisms discussed in the DPMS sections and the pyrolysis products identified by Py-GC/MS analysis of both homopolymers (Supplementary Materials Tables S1 and S2). According to the DPMS data, all copolymers develop CO_2_ in the onset of the thermal degradation process (about 400 °C), as well as a series of pyrolysis products typical of the ESES and ESDPA moieties, owing to the thermal processes which are involved (Scheme 2, Scheme 3 and Scheme 4) at the different temperatures. As an example, the Py-GC/MS data obtained for the P(ESES-co-ESDPA) 50/50 copolymer are discussed. Figure 12 shows the pyrograms recorded at 400, 500 and 600 °C, and the mass spectra data are summarized in Supplementary Materials Table S3. The mass spectra corresponding to each peak labeled in Figure 12 are shown in Supplementary MaterialsFigures S8–S10. At 400 °C, we observed the evolution of CO_2_, toluene and phenol, corresponding to peaks 1, 2 and 3, respectively, as displayed in Figure 12a. The pyrolysis products corresponding to peaks 8 and 10 are generated by the degradation processes of the diphenyl isobutyl moieties generated by decarboxylation of the DPA units. The diphenyl sulfone–phenyl ether observed at *m*/*z* 310 (peak 9) confirms the presence of phenyl ether–phenyl sulfone (ES) units along the copolymer chains. The other peaks in Figure 12a should be due to the EI fragmentation of the pyrolysis products with high mass. In accordance with the Py-GC/MS results obtained for the P(ESES) and P(ESDPA) homopolymers, at 500 °C, we determined several pyrolysis products (Figure 12b) due to the degradation of the ESES and the modified ESDPA sequences. As expected, the formation of SO_2_ (peak 1), phenol (peak 2) and diphenyl ether (peak 3) was revealed. The pyrolysis products corresponding to the peaks, 7, 8, 12 (Figure 12b and Supplementary Materials Table S3) are formed by the thermal degradation processes involving only the ESES sequences (Scheme 2 and Scheme 3). On the contrary, the products corresponding to the peaks 4, 9, 10, 11, 13a, 13b, 14, 18, 19 and 20 are generated by the degradation processes involving the modified ESDPA sequences (Scheme 4). Finally, at 600 °C (Figure 12c) was observed the formation of benzene (peak 2), toluene (peak 3), phenol (peak 4), diphenyl ether (peak 5) and dibenzofuran (peak 6). The products at *m*/*z* 184 (peak 8), 166 (fluorene, peak 7) and 242 (peak 10) are generated by the degradation processes due to the modified ESDPA units. The other peaks should be generated by EI (70eV) fragmentation of the high-mass pyrolysis products. The comparison of the pyrograms of the homopolymers and the copolymers recorded at 500 °C (Supplementary Materials Figure S11) allowed us to observe that the relative intensity of the peaks with retention times of about 13 min (peak 3) and 14.7–15.2 min (peak 4) depends on the ESES/ESDPA molar ratio in the samples.

## 4. Conclusions

Thermal degradation processes of the carboxylic acid functionalized P(ESES-co-ESDPA) copolymers with different molar composition were studied and compared with those of the corresponding homopolymers. All copolymers degrade in the temperature range from 370 to 650 °C, yielding a char residue ranging between 34 and 33 wt%, which decreases as the mol% of the diphenolic acid unit increases. All copolymers show a thermal stability slightly lower than that of the corresponding P(ESES) homopolymer. The EI (70 eV) DPMS data permitted us to identify the pyrolysis products and to deduce the possible thermal degradation mechanisms. DPMS data suggest that the initial thermal decomposition step of either the P(ESES-co-ESDPA) copolymers and the P(ESDPA) homopolymer is due to the decarboxylation process involving the pendant carboxyl groups belonging to the DPA units. The DPMS studies also reveal that, during the synthesis and the thermal treatment of the P(ESES-co-ESDPA) copolymers, the trans-etherification reaction occurs. Most of the pyrolysis products and the thermal degradation processes occurring at the different degradation temperatures (400, 500 and 600 °C) investigated were also confirmed by the stepwise Py-PCMS data. Our studies show the complementarity of the DPMS and Py-GC/MS investigations to obtain reliable information on the thermal-degradation processes which occur during the thermal treatment of the poly(ether sulfone)-based copolymers. Both mass spectrometry techniques provide fundamental information on the pyrolysis products formed and on the thermal-degradation mechanisms involved.

## Figures and Tables

**Table 1:** Thermogravimetric data of the P(ESES-co-ESDPA) copolymers and the corresponding homopolymers.

Samples		*T*_i_ (°C) ^(a)^	*T*_1/2_ (°C) ^(b)^	*T*_max 1_ (°C) ^(c)^	*T*_max 2_ (°C) ^(c)^	R (%) ^(d)^
1	P(ESES)	487	577	n.d.	566	35.4
2	P(ESES-co-ESDPA) 90:10	392	550	420	512	34.3
3	P(ESES-co-ESDPA) 80:20	391	547	425	510	34.5
4	P(ESES-co-ESDPA) 70:30	390	545	425	510	34.5
5	P(ESES-co-ESDPA) 50:50	395	519	435	493	34.1
6	P(ESES-co-ESDPA) 30:70	392	514	439	495	33.4
7	P(ESDPA)	382	515	437	495	32.4

^(a)^ Initial decomposition temperatures (5% weight loss); ^(b)^ half decomposition temperatures (50% weight loss); ^(c)^ temperature at the maximum rate of weight loss; ^(d)^ residue at 800 °C; n.d. not determined.

**Table 2:** Structural assignments of the molecular ions and fragments ions observed in the EI (70 eV) DPMS spectra.

Structure	n	M^+^	(M-64)	(M-66)	Samples ^(a)^				
					1	7	4	5	6
**H–[Ph–SO_2_–Ph–O]_n_–Ph–H**	1	310			+	-	-	-	-
	2	542	478						
**HO–[Ph–SO_2_–Ph–O]_n_–Ph–H**	0	94	262		+	-	-	-	-
	1	326	494						
	2								
**HO–[Ph–SO_2_–Ph–O]_n_–H**	1	250	186	184	+	-	-	-	-
	2	482	418						
**H–[Ph–SO_2_–Ph–O]_n_–H**	1	234	170	168	+	-	-	-	-
	2	466	402						
**HO–[Ph–SO_2_–Ph–O]_n_–Ph–OH**	0	110			+	-	-	-	-
	1	342	278	276					
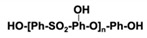	0	126			+	-	-	-	-
	1	358	294						
**H–Ph–SO_2_–Ph^+^**		217			+	-	-	-	-
**H–Ph–SO_2_^+^**		141			+	-	-	-	-
**HO–Ph–SO_2_^+^**		157			+	-	-	-	-
**Ph^+^**		77			+	-	-	-	-
**HO–Ph–O–Ph^+^**		185			+	-	-	-	-
**H–Ph–SO_2_–Ph–H**		217			+	-	+	+	+
**CH_2_=CH** **–** **^+^** **CH** **–** **CH_3_**		55			-	+	+	+	+
**SO_2_**		64			+	+	+	+	+
**CO_2_**		44			-	+	+	+	+
**HO–Ph–CH_2_^+^**		107			-	+	+	+	+
**HO–Ph–^+^CH–CH_3_**		121			-	+	+	+	+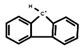		165			-	+	+	+	+
	
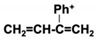		129			-	+	+	+	+
	
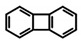		152			-	+	+	+	+
	
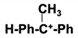		181			-	+	+	+	+
	
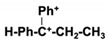		195			-	+	+	+	+
	
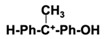		197			-	+	+	+	+
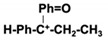		210			-	+	+	+	+
**HO–Ph–^+^CH–Ph–O–Ph–H**		275			-	+	+	+	+
**HO–Ph–^+^CH–Ph–O–Ph–SO_2_–Ph–OH**		431			-	+	+	+	+
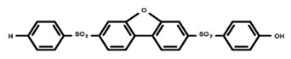		464			+	-	-	-	-
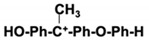		289			-	+	+	+	+
	
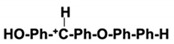		351			-	+	+	+	+
	
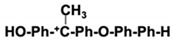		365			-	+	+	+	+
	
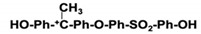		445			-	+	+	+	+
	
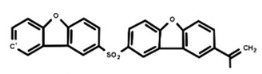		449			-	+	+	+	+
	
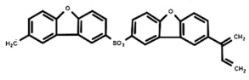		463			-	+	+	+	+
	
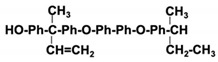		540			-	+	+	+	+
	
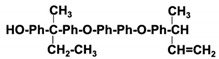		540			-	+	+	+	+
	
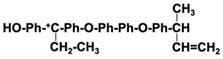		525			-	+	+	+	+
	
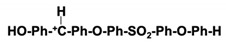		507			-	+	+	+	+
	
**H–Ph–^+^CH–Ph–O–Ph–SO_2_–Ph–O–Ph–H**		491			-	+	+	+	+
	
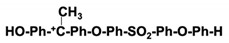		521			-	+	+	+	+
	
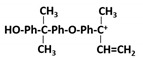		357			-	+	+	+	+
	
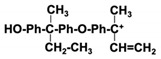		371			-	+	+	+	+
	
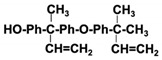		384			-	+	+	+	+
	

^(a)^ The symbol + indicates the presence of the ions; - denotes their absence.

**Table 3:** Identification of the product of pyrolysis formed at 500 °C.

Pyrolysis Product 500 °C	Peak Number	Mass (*m*/*z*)
SO_2_	1	64
Phenol	2	94
diphenyl ether	3	170
Dibenzofuran	4	168
4-hydroxy diphenyl sulfide	5	202
Pyrolysis product (fragmentation process Scheme 3)	7	246
Pyrolysis product (fragmentation process Scheme 2 and Scheme 3)	8	262
4-phenoxy-dibenzofuran	9	260
Pyrolysis product (fragmentation process Scheme 2 and Scheme 3)	10	278
Pyrolysis product (fragmentation process Scheme 2 and Scheme 3)	11	294
Pyrolysis product (fragmentation process Scheme 2)	13	326
Pyrolysis product (fragmentation process Scheme 2)	14	310
Pyrolysis product (fragmentation process Scheme 2)	15	338
Pyrolysis product (fragmentation process Scheme 2)	16	344
Pyrolysis product (fragmentation process Scheme 2 and Scheme 3)	17	338
Pyrolysis product (fragmentation process Scheme 3)	20	370

**Table 4:** Structural assignments of the most important pyrolysis products detected by stepwise Py-GC/MS of the poly(ether sulfones).

Ph-Structures of Pyrolysis Products	Mass	Ret. Time (min) (Where Present)			Temp. (°C)
		100:0	50:50	0:100	
**SO_2_**	64				400
		1.62	3.12	1.72	500
				1.53	600
**Toluene**	92		2.47	2.47	400
					500
			2.98	2.91	600
**Phenol**	94		4.44	4.99	400
		4.48	4.98	4.42	500
		4.48			600
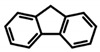	166				400
					500
			16.30	16.08	600
**Dibenzofuran**	168				400
		15.46			500
		15.13	15.39	15.77	600
**Ph–O–Ph**	170				400
		12.85	13.64	12.91	500
		13.01	13.53		600
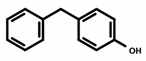	184				400
			14.99	14.69	500
			18.92	18.56	600
**9H, Fluorene, 9-phenyl**	242				400
					500
			22.20	22.22	600
**Ph–Ph–O–Ph**	246				400
		22.34	20.9	22.67	500
					600
**Ph–CH_2_–Ph–O–Ph**	260				400
		23.99	23.75	22.96	500
					600
HO–Ph–SO_2_–Ph–CH_2_–CH_3_	262				400
		22.62	26.03		500
					600
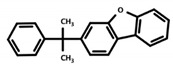	288				400
			26.76	24.33	500
					600
**Ph–SO_2_–Ph–O–Ph**	310		28.93	27.44	400
		27.42	27.46		500
					600
**CH_3_–Ph–O–Ph–SO_2_–Ph**	324			28.38	400
				28.35	500
					600
**Ph–SO_2_–Ph–O–Ph–CH_2_–CH_3_**	338				400
		27.58	27.59	29.22	500
					600
**Cl–Ph–SO_2_–Ph–O–Ph**	344		28.56	28.56	400
		28.63			500
					600
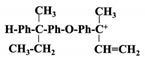	355				400
				27.42	500
					600
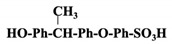	370				400
		31.81	31.77		500
					600
**HO–Ph–Ph–O–Ph–SO_2_–Ph**	402				400
		20.58			500
		21.14	20.31		600

## Supplementary Materials

The following are available online at https://www.mdpi.com/2073-4360/12/8/1810/s1, Figure S1a - ^1^H-NMR spectrum of the P(ESES-co-ESDPA) 50:50 copolymers., Figure S1b - FTIR spectrum of the P(ESES-co-ESDPA) 50:50 copolymers. Figure S2 - Pyrogram of the P(ESES) recorded at 400 °C and the EI (70 eV) mass spectra as a function of the retention time. Figure S3 - Py-GC mass spectra (70 eV) of the pyrolysis products of the P(ESES) pyrolyzed at 500 °C, as a function of the retention time. Figure S4 - Py-GC mass spectra of the pyrolysis products of the P(ESES) pyrolyzed at 600 °C, as a function of the retention time. Figure S5 - Py-GC mass spectra of the pyrolysis products of the P(ESDPA) sample pyrolyzed at 400 °C, as a function of the retention time. Figure S6 - Py-GC mass spectra of the pyrolysis products of the P(ESDPA) sample pyrolyzed at 500 °C, as a function of the retention time. Figure S7 - Py-GC mass spectra of the pyrolysis products of the P(ESDPA) sample pyrolyzed at 600 °C, as a function of the retention time. Figure S8 - Py-GC mass spectra of the pyrolysis products of the P(ESES-co-ESDPA) sample pyrolyzed at 400 °C, as a function of the retention time. Figure S9 - Py-GC mass spectra of the pyrolysis products of the P(ESES-co-ESDPA) sample pyrolyzed at 500 °C, as a function of the retention time. Figure S10 - Py-GC mass spectra of the pyrolysis products of the P(ESES-co-ESDPA) sample pyrolyzed at 600 °C, as a function of the retention time. Figure S11 - Pyrograms of the a) P(ESES), b) P(ESES-co-ESPA) 50:50, c) P(ESES-co-ESPA) 30:70 and d) P(ESDPA) samples, taken at 500 °C. Table S1 - Identification of the gaseous pyrolysis products in the stepwise Py-GCMS of P(ESES) sample at 500 °C and 600 °C. Table S2 - Identification of the gaseous pyrolysis products in the stepwise Py-GCMS of P(ESDPA) sample at 400 °C, 500 °C and 600 °C. Table S3 - Identification of the gaseous pyrolysis products in the stepwise Py-GCMS of the P(ESES-co-ESDPA) 50:50 random copolymer sample at 400 °C, 500 °C and 600 °C. Table S4: Some properties of the Poly(ether sulfone)s synthetized
