# Supplementary material for: Thermal Degradation Processes of Aromatic Poly(Ether Sulfone) Random Copolymers Bearing Pendant Carboxyl Groups
Source: Polymers (Basel). 2020 Aug 12;12(8):1810. doi: 10.3390/polym12081810 (PMC7464578; doi:10.3390/polym12081810)
Supplement: Supplementary file 1 [file polymers-12-01810-s001.zip › Figure Captions.docx]

Figure Captions:

Figure 1 - Overlay of thermogravimetric curves of P(ESDPA)-co-(ESES) copolymers and their homopolymers.

Figure 2 - Differential thermogravimetric (DTG) curves of the P(ESDPA)-co-(ESES) copolymers and the corresponding homopolymers. DTG curves are shifted vertically for clarity.

Figure 3 - TIC trace and single ion curves (SIC) of the ions at m/z 64, 326 and 217 evolved in the EI-DPMS of the P(ESES) homopolymer.

Figure 4 - EI-DPMS spectra of the P(ESES) sample recorded in the temperature ranges (a) 430-480 °C and (b) 480°C-500 °C.

Figure 5 - TIC trace and single ion curves (SIC) of the ions at m/z 44, 55, 64, 250, 351 and 507 evolved in the EI-DPMS of the P(ESDPA) homopolymer.

Figure 6 - EI-DPMS mass spectra of the P(ESDPA) polymer recorded in the temperature ranges (a) 380-430°C, (b) 430-480°C and (c) 480°C-500 °C.

Figure 7 - TIC trace and SIC curves of the ions at m/z 44, 64, 326, 351 and 507 recorded during the EI-DPMS of the random copolymer P(ESES)-co-(ESDPA) 50/50.

Figure 8 - EI-DPMS spectra of the P(ESES)-co-(ESDPA) random copolymers (a) 70/30, (b) 50/50 and (c) 30/70 recorded in the temperature range 480-500 °C.

Figure 9 - Evolution of the relative intensity of peak ions at m/z 507 and 326 as a function of the ES-ES %mol in the copolymers.

Figure 10 - Py-GCMS pyrograms P(ESES) detected at (a) 500, and (b) 600 °C.

Figure 11 - Py-GCMS pyrograms P(ESDPA) taken at (a) 400, (b) 500, and (c) 600 °C.

Figure 12 - Py-GCMS pyrograms P(ESES)-co-(ESDPA) 50:50 copolymer recorded at (a) 400, (b) 500, and (c) 600 °C.
