# Supplementary material for: Thermal Degradation Processes of Aromatic Poly(Ether Sulfone) Random Copolymers Bearing Pendant Carboxyl Groups
Source: Polymers (Basel). 2020 Aug 12;12(8):1810. doi: 10.3390/polym12081810 (PMC7464578; doi:10.3390/polym12081810)
Supplement: Supplementary file 1 [file polymers-12-01810-s001.zip › SI_Thermal Degradation processes of aromatic_06_08.docx]

**Supporting Information**

**Thermal Degradation Processes of Aromatic Poly(ether sulfone) Random Copolymers Bearing Pendant Carboxyl Groups**

**Sandro Dattilo,^1*)^ Concetto Puglisi^1)^, Emanuele F. Mirabella,^1)^ Angela Spina,^1)^ Andrea A. Scamporrino,^1)*^ Daniela C. Zampino,^1)^ Ignazio Blanco,^2)^ Gianluca Cicala,^1),2)^, Giulia Ognibene^2)^, Chiara Di Mauro^2)^, Filippo Samperi. ^1)^**

1) Institute for Polymers, Composites and Biomaterials, IPCB-CNR, Via Gaifami 18, 95126 Catania, Italy.

2) Department of Civil Engineering and Architecture, University of Catania, Viale Andrea Doria 6, 95125 Catania, Italy.

**Figure 1Sa** - ^1^H-NMR spectrum of the P(ESES-co-ESDPA) 50:50 copolymers.


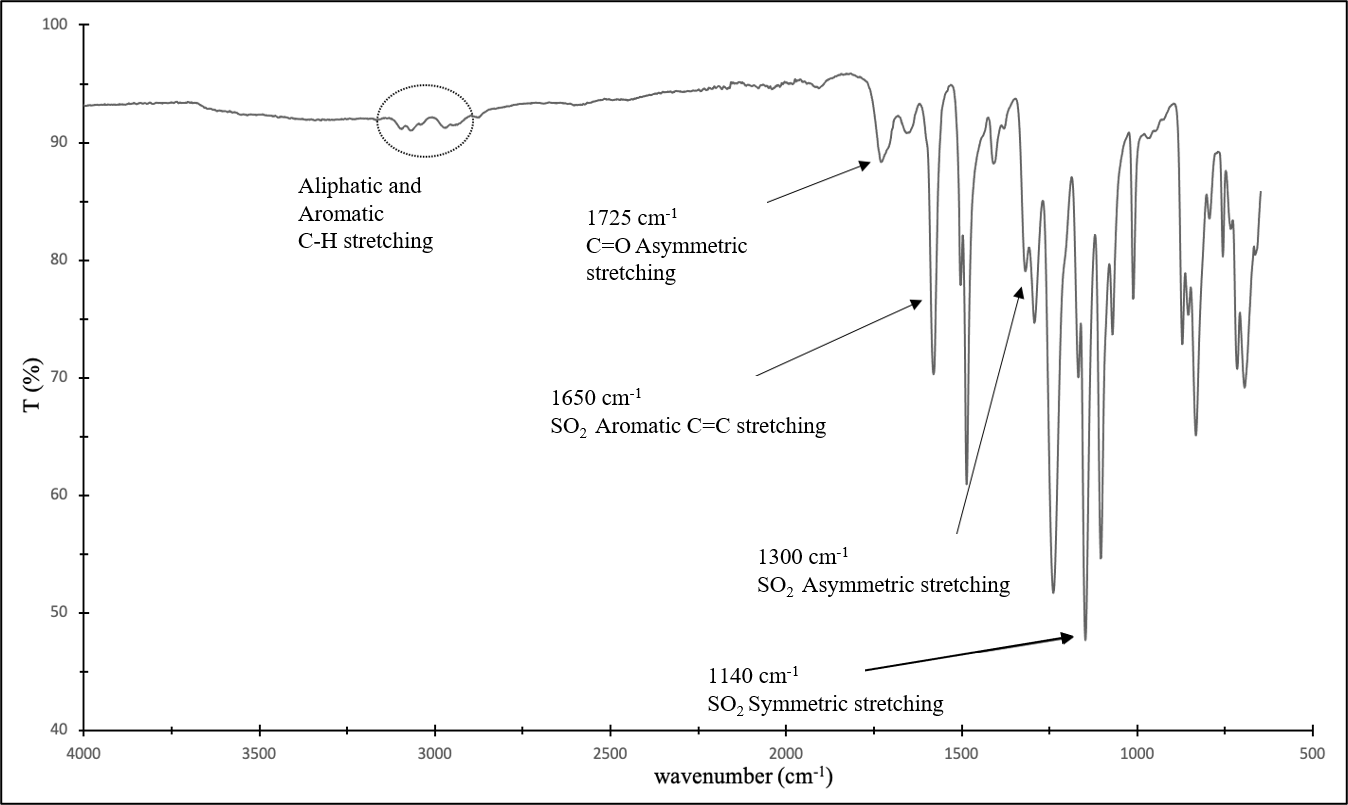
**Figure 1Sb** - FTIR spectrum of the P(ESES-co-ESDPA) 50:50 copolymers.

**Table 1S -** Identification of the gaseous pyrolysis products in the stepwise Py-GCMS of P(ESES) sample at 500°C and 600 °C.

| **Peak**  **N.** | **Pyrolysis**  **Product** | **Retention**  **Time (min)** | **%** | **Molar mass** | **Major Mass Fragments** |
| --- | --- | --- | --- | --- | --- |
| **500 °C** | | | | | |
| 1 | SO_2_ | 1.42 ÷ 3.26 | 16.40 | 64 | 64, 48 |
| 2 | Phenol | 4.43 ÷ 5.03 | 23.03 | 94 | 94, 66, 65, 55, 50, 39 |
| 3 | H-Ph-O-Ph-H | 13.30÷14.7 | 25.05 | 170 | 170, 141, 77, 51, 115, 39 |
| 4 | Dibenzofuran | 15.26÷15.67 | 0.5 | 168 | 168, 139, 94, 64, 39, 115 |
| 5 | 4-hydroxy diphenyl sulfide | 18.14÷18.75 | 2 | 202 | 186, 170, 94, 64, 77, 39, 168, 51, 141, 202, 115 |
| 6 | See Table 1 | 20.14÷21.03 | 2.5 | 402 | 94, 170, 184, 77, 64, 141, 39, 51, 168, 115, 246, 402, 217. 202, 129, 152, 229 |
| 7 | H-Ph-Ph-O-Ph-H | 22.29÷22.38 | 0.76 | 246 | 246, 232, 96, 94, 152, 139, 168, 115. 170, 77, 64, 51, 39, 217, 202, 184 |
| 8 | HO-Ph-Ph-O-Ph-H | 22.623 | 5.85 | 262 | 262, 246, 77, 141, 185, 51, 115, 129, 169, 217, 234, 202, 39, |
| 9 | 4-Phenoxy-Dibenzofuran | 23.997 | 6.25 | 260 | 260, 262, 231, 127, 77, 169, 139, 51, 203, 115, 101, 183, 89, 63 |
| 10 | HO-Ph-PH-O-Ph-H | 24.473 | 1.67 | 278 | 278, 185, 77 169, 141, 51, 115, 129, 152, 201, 262, 246 |
| 11 | H-Ph-SO_2_Ph-Ph-H | 25.547 | 0.90 | 294 | 262, 170, 294, 77, 141, 115, 51, 207, 94, 234, 39 |
| 12 | Fragments from pyrolysis products | 25.82÷25.90 | 2.3 |  | 262, 77, 157, 185, 258, 276,139, 128, 115, 51 |
| 13 | HO-Ph-SO_2_-Ph-O-Ph-H | 27.250 | 1.2 | 326 | 170, 326, 77, 115, 141, 62, 39, 207, 185, 262, 281 |
| 14 | Fragments from pyrolysis products | 27.420 | 4 | 310 | 310, 77, 185, 129, 115, 51, 141, 217, 169 |
| 15 | H-Ph-O-Ph-Ph-O-Ph-H  - 64 del 402 | 27.587 | 2.64 | 338 | 338, 77, 168, 139, 152, 202, 215, 51, 244, 227, 189, 115 |
| 16 | Cl-Ph-SO_2_-Ph-O-Ph-H | 28.627 | 0.4 | 344 | 344, 185, 77, 207, 115, 57, 129, 217, 141, 43, 281, 346, 111 |
| 16b | Fragments from pyrolysis products | 28.730 | 0.6 | 338 | 338, 207, 77, 57, 336, 139, 43, 215, 202, 55, 308, 152, 97, 253, 346, 357 |
| 17 | H-Ph-O-Ph-Ph-O-Ph-H | 29.490 | 1.6 | 338 | 338, 77, 152, 139, 202, 215, 217, 51, 207, 310, 57, 43, 281, 244, 233, 168, 115, 97 |
| 18 | Fragments from pyrolysis products | 30.073 | 1.85 | 338 | 338, 77, 261, 207, 233, 139, 152, 51, 281, 310, 189, 43 |
| 19 | Fragments from pyrolysis products | 31.423 | 0.5 | 326 | 326, 77, 57, 336, 207, 43, 69, 71, 81, 85, 115, 141, 186, 281, 97, 217, 253, 267, 231, 157, 129, 109, 97, 355, 405, 429 |
| 19b | Fragments from pyrolysis products | 31.517 | 0.45 |  | 326, 77, 207, 57, 352, 281, 185, 141, 43, 115, 129, 217, 253, 354, 405, 429 |
| 20 | H-Ph- Ph-Ph-SO_2_-Ph-H | 31.807 | 1.8 | 370 | 370, 77, 277, 171, 184, 115, 261, 51, 207, 139, 338, 326, 43, 57 |
| **600 °C** | | | | | |
| 1 | C_6_H_6_ | 2.21 | 7.2 | 78 | 78, 77, 52, 51, 50, 39 |
| 2 | Phenol + OH-Ph-OH | 4.48 | 29.2 | 94 | 94, 66, 65, 39, 110, 55, 50, 77 |
| 3 | H-Ph-Ph-OH | 13.013 | 6.5 | 170 | 154, 170, 141, 77, 51, 39, 115, 94 |
| 4 | Dibenzofuran | 15.133 | 7.1 | 168 | 168, 139, 154, 113 |
| 5 | H-Ph-SO_2_-Ph-O-Ph-Ph-H | 18.160 | 5 | 402 | 402, 185, 77, 217, 115, 141, 129, 170, 51, 261, 338, 39 |
| 6 | unknown products | 18.967 | 3.5 | 402 | 402, 77, 185, 217, 115, 170, 141, 129, 5, 152, 338, 261, 39 |
| 7 | unknown products | 20.427 | 36 | 402 | 402, 77, 185, 217, 115, 129, 141, 51, 168, 157, 338, 261, 39 |
| 8 | unknown products | 22.317÷22.913 | 0.8 | 402 | 246, 230, 77, 402, 115, 217, 185, 141, 152, 170, 51, 44, 129, 128, 39, 51, 281, 262, 428 |
| 9 | unknown products | 24.127 | 1.7 | 402 | 244, 402, 262, 215, 185, 77, 189, 141, 115, 95, 51, 129, 168, 39, 355, 281 |
| 10 | unknown products | 25.243÷27.243 | 1.7 |  | 207, 57, 258, 260, 281, 43, 68, 77, 402, 115, 185, 246, 191, 141, 355, 252, 327, 429 |
| 10 | unknown products | 27.153÷31.070 | 1.1 |  | 207, 57, 43, 69, 281, 71, 83, 97, 111, 133, 191, 253, 355, 327, 402, 429 |

**Figure 2S** - Pyrogram of the P(ESES) recorded at 400°C and the EI (70eV) mass spectra as a function of the retention time.

 ****

**Figure 3S** - Py-GC mass spectra (70 eV) of the pyrolysis products of the P(ESES) pyrolyzed at 500°C, as a function of the retention time.

**Figure 4S** - Py-GC mass spectra of the pyrolysis products of the P(ESES) pyrolyzed at 600°C, as a function of the retention time.

**Table 2S** - Identification of the gaseous pyrolysis products in the stepwise Py-GCMS of P(ESDPA) sample at 400°C, 500°C and 600 °C.

| **Peak**  **N.** | **Pyrolysis**  **Products** | **Retention**  **Time**  **(min)** | **%** | **Molar mass** | **Major Mass Fragments** |
| --- | --- | --- | --- | --- | --- |
| **400 °C** | | | | | |
| 1 | CO_2_ | 0.013 | 8.18 | 44 | 44, 45 |
| 2 | Toluene | 2.470 | 3.42 | 91 | 91,92, 44, 65, 39 |
| 3 | Phenol | 4.833÷5.147 | 4.0 | 94 | 94, 55, 44, 66, 39, 50, 72, 95 |
| 4 | unknown products | 22.940 | 2.7 |  | 129, 282, 77, 223, 115, 195, 51, 65, 94, 251, 285, 207, 165, 102, 39 |
| 5 | 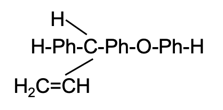 | 23.130 | 3.0 | 286 | 159, 75, 111, 161, 286, 131, 50, 94, 288, 50, 39 |
| 6a | Unknow | 23.707 | 6.2 | 282 | 130, 129, 282, 223, 115, 77, 91, 65, 51, 207 |
| 6b | Unknow | 23.787 |  | 282 | 253, 197, 115, 129, 77, 268, 223, 213, 43, 282, 141, 51, 94, 65, 39 |
| 7 | unknown products | 26.163 | 1.97 | 298 | 139, 298, 111, 207, 141, 75, 77, 300, 171, 94, 65, 39, 268, 281 |
| 8 | 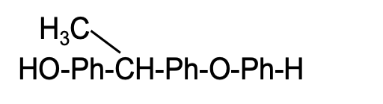 | 26.317 | 1.55 | 290 | 275, 290, 77, 141, 207, 181, 153, 139, 115, 94, 65, 51, 39, 306, 355 |
| 9 | unknown products | 26.863÷26.970 | 5.41 | 281 | 213, 207, 119, 91, 281, 300, 77, 65, 141, 165, 300, 39, 269, 355 |
| 10 | Ph-SO_2_-Ph-O-Ph | 27.440 | 2.10 | 310 | 310, 77, 185, 207, 129, 115, 141, 51, 217, 94, 39, 65, 73, 169, 253, 267, 355, 341, 327 |
| 11 | CH_3_-Ph-O-Ph-SO_2_-Ph | 28.383 | 1.62 | 324 | 324, 207, 281, 77, 91, 65, 199, 73, 143, 128, 231, 153, 253, 267, 125, 355, 341 |
| 12 | 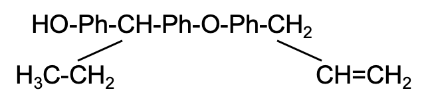 | 28.567 | 33.31 | 344 | 344, 185, 77, 115, 129, 217, 141, 141, 346, 75, 51, 169, 39, 281 |
| 13 | unknown products | 29.203 | 3.98 |  | 323, 338, 77, 207, 281, 154, 105, 51, 192, 65, 141, 253, 265, 355 |
| 14 | unknown products | 29.617 | 4.21 |  | 336. 207, 358, 77, 281, 91, 65, 155, 165, 199, 360, 231, 253, 128, 115, 51, 39. 405 |
| 15 | unknown products | 30.343÷30.457 | 2.55 |  | 351, 207, 281, 165, 77, 366, 73, 191, 253, 115, 133, 152, 141, 327, 52, 43, 405, 429, 479 |
| 16 | unknown products | 30.580 | 5.55 |  | 357, 372, 359, 207, 153, 105, 77, 374, 181, 111, 281, 75, 51, 39, 405 |
| 17 | unknown products | 32.093 | 10.12 |  | 384, 386, 207, 115, 165, 91, 111, 75, 281, 257, 181, 141, 65, 39, 369, 355, 429 |
| **500 °C** | | | | | |
| 1 | SO_2_ _100%_ | 1.520÷1.92 | 11.0 | 64 | 64, 48, |
| 2 | Phenol _93%_ | 4.293 ÷ 4.557 | 50.10 | 94 | 94, 66, 65, 39 |
| 3 | Ph-O-Ph | 12.910 | 2.90 | 170 | 170, 141, 142, 77, 51, 169, 115, 39, 94, 65 |
| 4 | 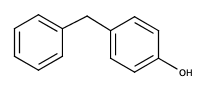_88%_ | 14.69 | 6.90 | 184 | 184, 91, 77, 65, 51, 141, 39, 155, 115, 169, 128, 63 |
| 5 | 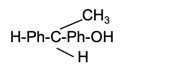 | 16.130 | 0.90 | 198 | 183, 198, 77, 51, 184, 186, 155, 65, 39, 94, 168, 115, 105 |
| 6 | 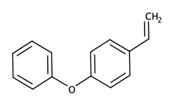 | 16.730 | 0.80 | 196 | 196, 167, 77, 51, 94, 168, 39, 65, 91, 153, 184, 141, 115 |
| 7 | 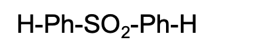 | 20.097÷20.677 | 1.80 | 218 | 202, 184, 77. 51,39, 141, 170, 128, 94, 115, 65, 157, 218, 230, 246 |
| 8 | 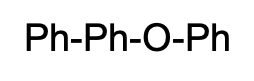 | 22.673 | 0.30 | 246 | 246, 115, 152, 141, 77, 181, 202, 217, 192, 51, 39, 94, 65, 258 |
| 9 | 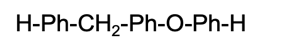 | 22.963 | 1.14 | 260 | 167, 260, 165, 152, 77, 51, 115, 183, 128, 200, 91, 65, 39, 91, 246 |
| 10 | 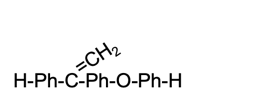 | 23.717 | 0.55 | 272 | 272, 179, 165, 77, 152, 51, 257, 115, 139, 39, 65, 91, 228, 200, 102 |
| 11 | 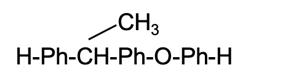 | 23.903÷24.110 | 0.55 | 274 | 262, 274, 181, 165, 77, 166, 115, 51, 141, 152, 169, 51, 260, 65, 139, 39, 207, 200 |
| 11b | 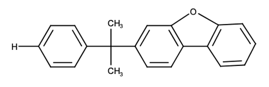 | 24.33 |  | 288 | 273, 234, 141, 77, 126, 288, 94, 115, 207, 180, 260 |
| 12 | unknown products | 24.437 | 1.97 | 278 | 278, 77, 185, 141, 51, 169, 234, 201, 126, 115, 39, 152, 94, 273, 258 |
| 13 | 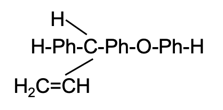 | 24.723 | 0.16 | 286 | 286, 178, 193, 165, 77, 141, 234, 51, 126, 115, 287, 271, 39, 65, 109, 152, 208, 253 |
| 14 | unknown products | 25.450 | 0.84 | 292 | 292, 185, 169, 293, 141, 65, 77, 91, 51, 153, 201, 128, 115, 39, 258, 276, 215, 109 |
| 15 | 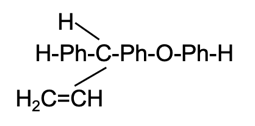 | 25.917 | 3.13 | 286 | 276, 183, 286, 165, 77, 181, 152, 153, 51, 115, 262, 141, 139, 107, 39, 89, 65, 63, 209 |
| 16 | 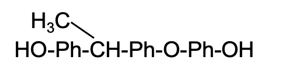 | 26.277 | 1.0 | 306 | 275, 290, 77, 181, 306, 169, 152, 51, 115, 65, 29, 197, 165, 91 |
| 17 | 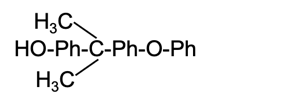 | 26.66 | 2.88 | 304 | 288, 195, 165, 177, 77, 273, 152, 51, 65, 39, 139, 115, 91, 128, 245, 207, 304 |
| 18 | 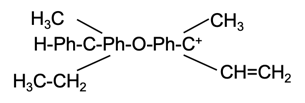 | 27.423 | 0.64 | 355 | 310, 77, 185, 115, 51, 129, 141, 207, 217, 281, 157, 39, 94, 139, 65, 355, 327 |
| 19 | 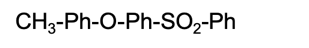 | 28.35 | 2.30 | 324 | 324, 199, 91, 77, 65, 325, 143, 231, 128, 153, 155, 171, 51, 115, 281, 39, 207, 168 |
| 20 | 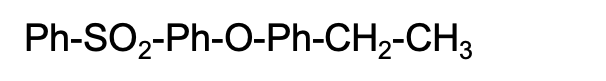 | 29.22 | 0.34 | 338 | 322, 338, 207, 77, 281, 153, 288, 181, 51, 165, 152, 288, 253, 105, 73, 265, 355, 39, 405, 429 |
| 21 | 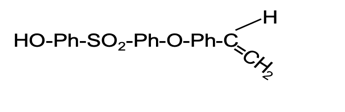 | 30.040 | 2.70 | 352 | 259, 352, 165, 77, 181, 153, 51, 115, 207, 89, 141, 338, 281, 39 |
| 22 | 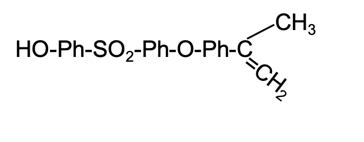 | 30.357 | 2.17 | 366 | 351, 366, 165, 77, 153, 207, 178, 51, 115, 281, 257, 229, 139, 91, 39 |
| 23 | 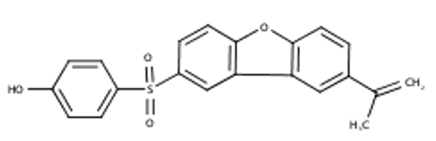 | 30.890 | 4.0 | 364 | 364, 178, 271, 165, 77, 51, 349, 153, 228, 207, 19, 39 |
| 24 | 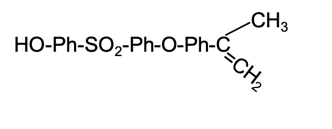 | 31.297 | 0.28 | 366 | 366, 207, 165, 259, 281, 273, 77, 181, 152, 91, 65, 253, 355, 327, 115, 51, 73, 43, 430, 405 |
| 25 | unknown products | 31.653 | 0.12 | 380 | 365, 207, 380, 165, 281, 77, 152, 253, 73, 91, 65, 327, 472, 429 |
| 26 | unknown products | 32.310 | 0.51 | 378 | 378, 178, 207, 285, 281, 77, 271, 91, 65, 73, 152, 253, 43, 355, 327, 115, 228, 430 |
| **600 °C** | | | | | |
| 1 | CO_2_ + SO_2_ | 1.350÷1.727 | 6.6 | 44 | 44, 64 |
| 2 | Benzene | 1.940÷2.373 | 13.1 | 78 | 78, 44, 77, 51, 50, 52, 39, 63 |
| 3 | Toluene | 2.383÷3.453 | 14.0 | 91 | 78, 44, 91, 92, 77, 51, 39, 49 |
| 4 | unknown products | 4.410÷5.623 | 11.03 |  | 94, 66, 39, 44, 110, 78, 51, 50, 39, 52, 63, 65 |
| 5 | unknown products | 13.067÷13.430 | 3.34 |  | 154, 153, 170, 141, 77, 51, 44, 39, 115, 94, 63, 128 |
| 6 | unknown products | 13.550÷13.997 | 2.90 |  | 154, 153, 167, 168, 152, 170, 165, 77, 44, 39, 51, 141, 65, 94, 115 |
| 7 | Dibenzofuran | 15.3 | 11.27 | 168 | 168, 139, 154, 113, 84, 63, 91, 39, 44 |
| 8 | Dibenzofuran | 15.777 | 3.56 | 168 | 168, 139, 154, 113, 84, 63, 91, 39, 44 |
| 9 | Fluorene | 16.087 | 7.05 | 166 | 166, 165, 168, 139, 154, 115, 82, 63, 39, 74, 70, 44 |
| 10 | 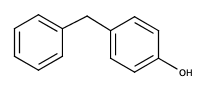 | 18.560 | 4.17 | 184 | 184, 139, 152, 92, 75, 165, 158, 86, 63, 50, 44, 39 |
| 11 | unknown products | 18.733÷18.993 | 5.01 |  | 178, 184, 152, 180, 176, 139, 76, 89, 63, 44 |
| 12 | 9H, Fluorene, 9-phenyl | 22.22 | 4.53 | 242 | 242, 241, 165, 239, 226, 213, 202, 184, 139, 119, 44, 77, 63, 39 |
| 13 | unknown products | 24.230÷24.553 | 12.56 |  | 244, 207, 281, 44, 73, 165, 200, 215, 77, 171, 139, 115, 107, 94, 122, 189, 218, 478, 429, 402, 355, 327, 258, 252 |
| 14 | unknown products | 28.303÷28.937 | 1.04 |  | 207, 281, 73, 253, 191, 416, 355, 327, 133, 165, 96, 57, 77, 44, 115 |

**Figure 5S** - Py-GC mass spectra of the pyrolysis products of the P(ESDPA) sample pyrolyzed at 400°C, as a function of the retention time.

**Figure 6S** - Py-GC mass spectra of the pyrolysis products of the P(ESDPA) sample pyrolyzed at 500°C, as a function of the retention time.

**Figure 7S** - Py-GC mass spectra of the pyrolysis products of the P(ESDPA) sample pyrolyzed at 600°C, as a function of the retention time.

**Table 3S -** Identification of the gaseous pyrolysis products in the stepwise Py-GCMS of the P(ESES-co-ESDPA) 50:50 random copolymer sample at 400°C, 500°C and 600 °C.

| **Peak**  **N.** | **Pyrolysis**  **Product** | **Retention**  **Time (min)** | **Molar mass** | **%** | **Major Mass Fragments** |
| --- | --- | --- | --- | --- | --- |
| **400 °C** | | | | | |
| 1 | CO_2_ | 0.477÷0.773 | 44 | 33.35 | 44, 45, 40 |
| 2 | Toluene | 2.470 | 92 | 0.96 |  |
| 3 |  | 2.670 |  | 3.76 |  |
| 4 | Phenol | 4.443 | 94 | 38.35 | 94, 66, 65, 63, 39, 95, 55, 50 |
| 5 | unknown products | 14.613 | 220 | 1.58 | 205, 220, 177, 57, 145, 105, 91. 41, 161, 89, 133, 121, 81, 77 |
| 6 | unknown products | 25.733÷26.017 |  | 3.23 | 141, 268, 109, 65, 39, 75, 270, 75, 81, 93, 281, 429, 355, 327 |
| 7 | unknown products | 26.137 |  | 2.20 | 139, 298, 111, 300, 75, 171, 45, 141, 268, 207, 77, 39, 401, 377, 355, 327, 312 |
| 8 | 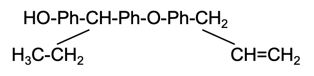 | 28.567 | 344 | 4.10 | 344, 185, 77, 115, 129, 217, 141, 346, 75, 111, 51, 207, 281, 139, 39, 429, 405, 355 |
| 9 | 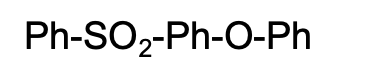 | 28.937 | 310 | 11.75 | 310, 171, 139, 77, 45, 123, 111, 69, 207, 281, 231, 253, 355, 490, 475, 429, 405, 377, 355, 327 |
| 10 | 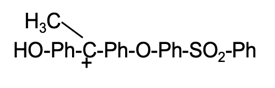 | 29.920÷34.090 | 429 | 0.72 | 207, 281, 57, 43, 55, 69, 71, 73, 97, 83, 81, 85, 95, 253, 191, 355, 327, 165, 133, 429, 405, 341, 500, 461, 479 |
| **500 °C** | | | | | |
| 1 | SO_2_ | 1.633÷2.980 | 64 | 11.30 | 64, 48, 66 44 |
| 2 | Phenol | 4.533÷5.433 | 94 | 44.35 | 94,66, 65, 39,40 |
| 3 | Ph-O-Ph | 12.867÷14.420 | 170 | 16.46 | 170, 141, 142, 77, 51, 161, 115, 94, 39, 65 |
| 4 | 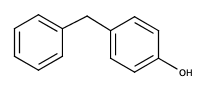 | 14.993 | 184 | 1.12 | 184, 91, 94, 170, 66, 141, 77, 51. 39, 115, 156, 168, 165, 153, 128, 107 |
| 5 | unknown products | 15.770÷16.993 |  | 0.64 | 168, 94, 66, 139, 170, 184, 39, 186, 51, 77, 91, 115, 142, 152 |
| 6 | 4 hydroxy diphenyl sulfide | 18.220 | 202 | 1.41 | 202, 94, 77, 141, 173, 51. 39, 170, 168, 184, 115, 173, 109 |
| 7 | Ph-Ph-O-Ph | 20.5÷ 21.3 | 246 | 1.63 | 202, 94, 170, 184, 66, 39, 77, 141, 65, 51, 246, 152, 139, 128, 44, 218, 63, 229 |
| 8 | HO-Ph-Ph-O-Ph-H | 22.717 | 262 | 1.42 | 246, 141, 77, 262, 115, 202, 181, 170, 51, 217, 94, 39, 63, 281, 127, 102 |
| 9 | 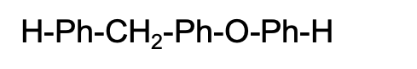 | 23.75 | 260 | 0.33 | 167, 260, 246, 77, 94, 152, 115, 51, 39, 141, 184, 66, 218, 202, 281, 75, 63, 44, 207, 91, 128, 39 |
| 10 | 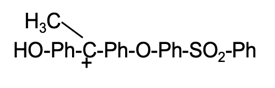 | 23.713÷23.840 | 429 | 0.46 | 272, 77, 94, 200, 179, 165, 207, 171, 152, 141, 115, 66, 44, 39, 51, 207, 260, 281, 246, 102, 217, 355, 429 |
| 11 | 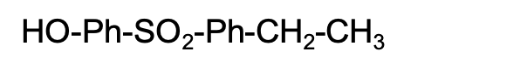 | 24.027÷24.087 | 262 | 2.0 | 262, 260, 169, 141, 77. 128, 115, 139, 127, 51, 231, 39, 44, 155, 94, 207, 102, 65, 185, 283, (474, 451, 402, 355) |
| 12 | HO-Ph-PH-O-Ph-H | 24.443 | 278 | 5.12 | 278, 185, 77, 169, 184, 281, 141, 51, 175, 201, 152, 139, 115, 65, 262, 246, 217 |
| 13a | 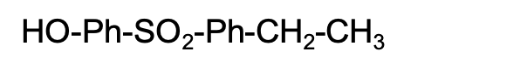 | 25.940÷26.133 | 262 | 1.25 | 252, 207, 77, 281, 276, 263, 185, 157, 51, 39, 234, 139, 141, 118, 128, 94, 57, 45, 46, 253, 355, 327, 419, 402 |
| 13b | 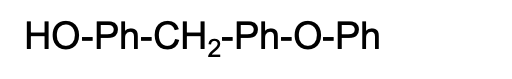 | 26.180÷26.273 | 276 | 1.30 | 272, 262, 207, 77, 171, 281, 294, 139, 51, 94, 157, 39, 152, 312, 365, 347, 253, 355, 327, 341, 429 |
| 14 | 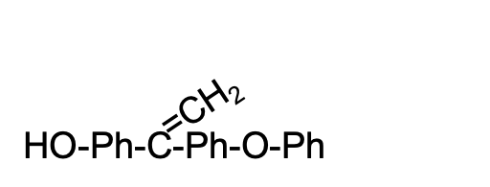 | 26.697÷26.823 | 288 | 1.88 | 288, 207, 195, 165, 77, 177, 281, 262, 152, 139, 115, 57, 43, 273, 253, 94, 355, 327, 429 |
| 15 | 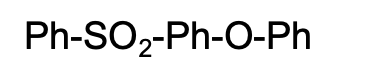 | 27.420÷27.500 | 310 | 0.87 | 310,77, 185, 207, 115, 129, 51, 141, 281, 217, 51, 43, 39, 253, 169, 355, 327, 429, 405 |
| 16 | 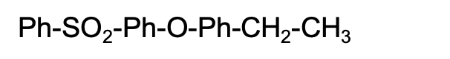 | 27.533÷27.660 | 338 | 1.14 | 338, 294, 207, 77, 281, 185, 310, 51, 168, 139, 115, 43, 253, 94, 217, 262, 265, 355, 327, 429, 405 |
| 17 | unknown products | 29.003÷29.073 |  | 0.63 | 207, 281, 57, 43, 55, 253, 191, 73, 69, 97, 355, 327, 405, 429 |
| 18 | 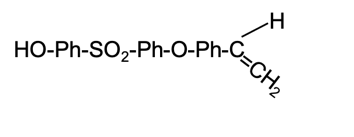 | 30.007÷30.113 | 352 | 1.11 | 207, 338, 281, 77, 55, 43, 259, 352, 267, 263, 312, 354, 405, 429 |
| 19 | 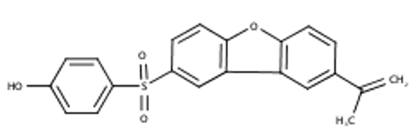 | 30.833÷30.913 | 364 | 2.46 | 364, 178, 271, 207, 165, 77, 281, 57, 71, 43, 281, 253, 349, 152, 139, 429, 405 |
| 20 | 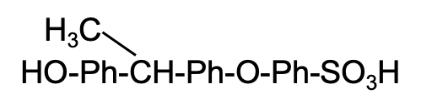 | 31.713÷31.833 | 370 | 3.11 | 370, 207, 77, 277, 184, 171, 281, 261, 115, 57, 51, 43, 139, 338, 355, 429, 405 |
| **600 °C** | | | | | |
| 1 | Fragmentation of compounds | 1.307÷1.697 | 44 | 1.15 | 44, 64 |
| 2 | Benzene _91%_ | 2.053÷2.560 | 78 | 2.84 | 78, 44, 77, 51, 50, 39 |
| 3 | Toluene | 2.737÷3.243 | 91 | 1.18 | 78, 91, 44, 92, 77, 51, 50, 39, 52, 63, 69 |
| 4 | Phenol _91%_ | 4.527÷5.317 | 94 | 19.94 | 94, 66, 65, 39, 75, 110, 95, 55, 50 |
| 5 | 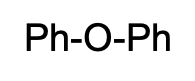 | 13.273÷13.800 | 170 | 4.90 | 154, 170, 153, 152, 141, 77, 51, 94, 44, 39, 143, 115, 66 |
| 6 | Dibenzofuran | 15.390 | 168 | 1.05 | 168, 139, 154, 113, 91, 84, 63, 44, 91, 77, 63, 69, 57, 39 |
| 7 | 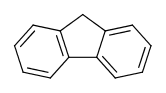 | 16.043÷16.570 | 166 | 1.90 | 166, 167, 168. 139, 115, 186, 185, 44, 82, 152, 74, 63, 51 |
| 8 | 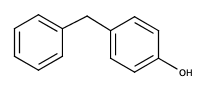 | 18.417÷19.427 | 184 | 5.60 | 184, 170, 152, 139, 178, 185, 44, 115, 141, 92, 79, 63, 39 |
| 9 | 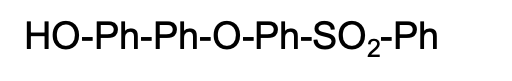 | 20.307 | 402 | 1.17 | 402, 170, 77, 185, 217, 115, 141. 44, 403, 129, 139, 168, 51, 152, 178, 39 |
| 10 | 9H, Fluorene, 9-phenyl | 22.197 | 242 | 1.13 | 242, 241, 239, 165, 184, 163, 44, 77, 207, 202, 139, 120, 115, 402 |
| 11 | unknown products | 24.043÷25.143 |  | 2.10 | 244, 207, 281, 200, 44, 77, 171, 75, 215, 115, 139, 165, 57, 55, 185, 402, 95, 355 |
| 12 | unknown products | 25.450÷27.780 |  | 24.70 | 207, 281, 57, 73, 43, 253, 258, 260, 191, 97, 133, 355, 327, 165, 147, 111, 402, 429, 479 |
| 13 | unknown products | 28.043÷30.680 |  | 34.40 | 207, 281, 57, 43, 73, 71, 69, 55, 253, 191, 97, 95, 43, 41, 355, 327, 133, 105, 165, 405, 429 |

**Figure 8S** - Py-GC mass spectra of the pyrolysis products of the P(ESES-co-ESDPA) sample pyrolyzed at 400°C, as a function of the retention time.

**Figure 9S** - Py-GC mass spectra of the pyrolysis products of the P(ESES-co-ESDPA) sample pyrolyzed at 500°C, as a function of the retention time.

**Figure 10S** - Py-GC mass spectra of the pyrolysis products of the P(ESES-co-ESDPA) sample pyrolyzed at 600°C, as a function of the retention time.

**Figure 11S** - Pyrograms of the a) P(ESES), b) P(ESES-co-ESPA) 50:50, c) P(ESES-co-ESPA) 30:70 and d) P(ESDPA) samples, taken at 500 °C.


**Table 4S:** Some properties of the Poly(ether sulfone)s synthetized

| **Sample** | **Feed Ratio (Mol Fraction)**  **DCDPS DHDPS DPA** | **Molar Composition (%_mol_) ^1^ ESES/ESDPA**  **^1^H-NMR** | **Mn (g/mol)^2^**  **(^1^H-NMR)** | **(Mw/Mn) ^3^** | **Yield^4^**  **(%)** |
| --- | --- | --- | --- | --- | --- |
| **1** | 1.0 1.0 0.0 | 100/0 | 7900 | 18200/8300 | 93.2 |
| **2** | 1.0 0.9 0.1 | 88/12 | 7000 | 15000/7150 | 94.1 |
| **3** | 1.0 0.8 0.2 | 77/23 | 7100 | 15700/7300 | 92.7 |
| **4** | 1.0 0.7 0.3 | 68/32 | 8800 | 19800/9000 | 92.5 |
| **5** | 1.0 0.5 0.5 | 51/49 | 6600 | 15000/6850 | 94.6 |
| **6** | 1.0 0.3 0.7 | 32/68 | 7200 | 15800/7400 | 93.4 |
| **7** | 1.0 0.0 1.0 | 0/100 | 6000 | 13900/6150 | 91.2 |

^1)^ Calculated through ^1^H analysis [37]. ^2)^Number Average Molar Mass determined by ^1^H-NMR. ^3)^ Calculated by SEC analysis, using DMF as eluent and applying calibration curve build with polystyrene narrow standards. ^4)^ Calculated after purification procedure.
